# Characterization of Key Aroma Compounds of Soy Sauce-like Aroma Produced in Ferment of Soybeans by *Bacillus subtilis* BJ3-2

**DOI:** 10.3390/foods13172731

**Published:** 2024-08-28

**Authors:** Qibo Tan, Yongjun Wu, Cen Li, Jing Jin, Lincheng Zhang, Shuoqiu Tong, Zhaofeng Chen, Li Ran, Lu Huang, Zeyan Zuo

**Affiliations:** 1 Key Laboratory of Plant Resource Conservation and Germplasm Innovation in Mountainous Region (Ministry of Education), College of Life Sciences/Institute of Agro-Bioengineering, Guizhou University, Guiyang 550025, China; qibotan@outlook.com (Q.T.); cenli@gzu.edu.cn (C.L.); jjin3@gzu.edu.cn (J.J.); zhanglc201312@163.com (L.Z.); m18786120419@163.com (S.T.); zfchen2024@outlook.com (Z.C.); rainnie0722@outlook.com (L.R.); hl.2025@outlook.com (L.H.); 2 Guizhou Institute of Products Quality Inspection & Testing, Guiyang 550016, China; 15285996397@163.com

**Keywords:** *Bacillus subtilis*, soy sauce-like aroma, GC-O-MS, key aroma compounds, fermentation

## Abstract

Fermented soybeans are popular among many for their rich soy sauce-like aroma. However, the precise composition of this aroma remains elusive, with key aroma compounds unidentified. In this study, we screened the candidate genes *ilvA* and *serA* in BJ3-2 based on previous multi-omics data, and we constructed three mutant strains, BJ3-2-*ΔserA*, BJ3-2-*ΔilvA*, and BJ3-2-*ΔserAΔilvA*, using homologous recombination to fermented soybeans with varying intensities of soy sauce-like aroma. Our objective was to analyze samples that exhibited different aroma intensities resulting from the fermented soybeans of BJ3-2 and its mutant strains, thereby exploring the key flavor compounds influencing soy sauce-like aroma as well analyzing the effects of *ilvA* and *serA* on soy sauce-like aroma. We employed quantitative descriptive sensory analysis (QDA), gas chromatography–olfactometry–mass spectrometry (GC-O-MS), relative odor activity value analysis (rOAV), principal component analysis (PCA), orthogonal partial least squares-discriminant analysis (OPLS-DA), and partial least squares regression analysis (PLSR). QDA revealed the predominant soy sauce-like aroma profile of roasted and smoky aromas. GC-MS detected 99 volatile components, predominantly pyrazines and ketones, across the four samples, each showing varying concentrations. Based on rOAV (>1) and GC-O, 12 compounds emerged as primary contributors to soy sauce-like aroma. PCA and OPLS-DA were instrumental in discerning aroma differences among the samples, identifying five compounds with VIP > 1 as key marker compounds influencing soy sauce-like aroma intensity levels. Differential analyses of key aroma compounds indicated that the mutant strains of *ilvA* and *serA* affected soy sauce-like aroma mainly by affecting pyrazines. PLSR analysis indicated that roasted and smoky aromas were the two most important sensory attributes of soy sauce-like aroma, with pyrazines associated with roasted aroma and guaiacol associated with smoky aroma. In addition, substances positively correlated with the intensity of soy sauce-like aroma were verified by additional experiments. This study enhances our understanding of the characteristic flavor compounds in soy sauce-like aroma ferments, provides new perspectives for analyzing the molecular mechanisms of soy sauce-like aroma formation, and provides a theoretical framework for the targeted enhancement of soy sauce-like aroma in various foods.

## 1. Introduction

Soy sauce-like aroma is widely appreciated in various foods for its lightness, delicacy, richness, and longevity [1,2]. It plays a crucial role in traditional foods such as soy sauce, soybean paste, tempeh, and saucy white wine, influencing their acceptability [3,4]. To understand its formation, researchers have analyzed soy sauce-like aroma using various analytical tools with these foods as subjects.

Fan et al. investigated the volatile components of soy sauce-like aroma in Lang and Maotai sauce-type liquors, highlighting compounds like 2,3,5-trimethyl-6-ethylpyrazine, 3-methylbutanoic acid, 2,3,5-trimethylpyrazine, 2,3,5,6-tetramethylpyrazine, 4-methylguaiacol, and others [5,6,7]. Lin et al. identified 14 compounds that accurately mimicked the aroma of Pixian bean sauce, including 2,3,5,6-tetramethylpyrazine, 2,5-dimethylpyrazine, furfural, and 4-ethyl-2-methoxyphenol, using SPME-GC-O/MS and aroma recombination, emphasizing pyrazines, furans, and aldehydes as key contributors to soy sauce-like aroma [8]. In 2024, Duan et al. demonstrated that 2,3,5-trimethylpyrazine, 3-hydroxy-2-butanone, and several other substances may be critical flavor compounds in Langjiu’s soy sauce-like aroma through recombination and omission tests [9]. Pyrazines, 3-methylbutyric acid, maltol, and methoxyphenol are found in higher concentrations in cheonggukjang, contributing to its characteristic sauce aroma [1]. Recently, Zhao et al. proposed a new theory based on their study of volatiles in three Japanese soy sauces, linking amino acids closely to the production of volatile compounds like pyrazines [10]. These studies collectively indicate that soy sauce-like aroma is not attributed to a single substance but arises from the synergistic action of various aroma compounds [11,12]. Despite these findings, the complete composition of soy sauce-like aroma remains incompletely elucidated.

It has been proposed that beans and flour can undergo fermentation by microorganisms to yield a rich soy sauce-like aroma [13]. *Bacillus subtilis* is closely associated with the production of soy sauce-like aroma [14], with wide-ranging applications in the fermentation industry and beyond [15,16]. Transcriptome, proteome, and metabolome analyses have further elucidated the differential expression of genes and metabolic pathways, particularly in amino acid metabolism under varying temperatures, suggesting their role in soy sauce-like aroma accumulation [17]. Based on BJ3-2’s capacity to generate soy sauce-like aroma from fermented soybeans at 53 °C and supported by multi-omics data, *Bacillus subtilis* BJ3-2 serves as an ideal model for investigating the molecular mechanisms and key aroma compounds of soy sauce-like aroma. The flavor candidate genes *ilvA* and *serA* were screened by combined transcriptomic, proteomic, and metabolomic analyses of BJ3-2 in relation to those that are likely to be associated with the soy sauce-like aroma. In the KEGG pathway, *ilvA* is the gene encoding threonine dehydratase, and *serA* is the gene encoding D-3-phosphoglycerate dehydrogenase. The metabolic pathways involved in both *ilvA* and *serA* are the glycine, serine, and threonine metabolic pathways [18,19]. It has been shown that the metabolism of amino acids is closely related to the formation of the soy sauce-like aroma [20]. And it has been reported that the metabolism of serine and threonine is highly related to the formation of pyrazines [21], so the knockout of *ilvA* and *serA* may lead to pyrazines and other aroma compounds, thus affecting the soy sauce-like aroma. Thus, we could systematically analyze aroma-active compounds that produce soy sauce-like aroma in soybeans fermented at 53 °C using wild-type strains of Bacillus subtilis BJ3-2 and mutant strains of *ilvA* and *serA* to elucidate the formation of soy sauce aroma in solid-state fermentation. For flavor analysis, GC-MS combined with a headspace solid-phase microextraction technique (HS-SPME technique) can be used to concentrate analytes in samples by extracting phase-encapsulated fiber heads and then enable the effective separation and identification of volatile compounds across a wide range of foods in GC-MS [22]. Additionally, the integration of GC-O, quantitative analysis, and various data analysis methods offers insights into the intricate relationships between sensory perceptions and differential aroma substances among different samples [23].

In this study, the aim of this study was (1) to screen soy sauce-like aroma candidate genes through multi-omics data analysis and (2) to obtain mutants with significantly altered soy sauce-like aroma by knocking out soy sauce-like aroma candidate genes, which produce a more pronounced soy sauce aroma, so as to better analyze the relationship between sensory attributes and soy sauce flavor aroma compounds; (3) Simulated fermentations were conducted using these mutant strains and BJ3-2 to produce soy sauce-like aroma, followed by sensory evaluations. (4) Volatile compounds from the fermentations exhibiting soy sauce-like aroma were analyzed using GC-MS with a semi-quantitative approach. (5) Characteristic aroma compounds of soy sauce-like aroma were identified using rOAV in conjunction with GC-O. (6) The variability among samples with different soy sauce-like aroma intensities was further analyzed using PCA and OPLS-DA. (7) The correlation between key aroma compounds and sensory attributes was analyzed and validated by partial least squares regression analysis (PLSR) and additive experiments. (8) The effect of knockout candidate genes on the formation of soy sauce-like aroma compounds was analyzed by the results of the KEGG metabolic pathway analysis of candidate genes and the statistical difference analysis of key aroma substances. In this study, we characterized for the first time the aroma profile of soy sauce-like aroma in fermented samples containing *Bacillus subtilis* BJ3-2 and its different mutants using multi-omics data, gene knockout, and headspace solid-phase microextraction (HS-SPME) methods combined with gas chromatography–mass spectrometry (GC-O-MS), rOAV, and an aroma addition experiment. These investigations focused on key volatiles enhancing soy sauce-like aroma, deepening our understanding of the characteristic aroma compounds in foods with soy sauce-like aroma, gaining insights into the key aroma compounds that produce a soy sauce-like aroma during the fermentation of soybeans by Bacillus subtilis, and providing theoretical insights for the targeted enhancement of soy sauce-like aroma in food products. These data also provide new strategies for screening soy sauce-like aroma candidate genes and modifying fermentation flavors through genetic engineering.

## 2. Materials and Methods

### 2.1. Strains and Vectors

*Bacillus subtilis* BJ3-2 is a patented strain from our laboratory (Patent No. 201110023795.4; link) [24]. Escherichia coli DH5α and pUC18 vectors were purchased from TaKaRa (Dalian, China). The pHT-01cas9-p43 vector was obtained from Biomics Biotechnology (Nantong, China), and the pMarA vector was a generous gift from China Agricultural University.

### 2.2. Chemicals

Retention indices (RI) were calculated using n-alkane standards (C7-C30) (Supelco, Bellefonte, PA, USA). The internal standard sec-octanol (99%) was purchased from Aladdin Industries Corporation (Shanghai, China). Reference standards were sourced as follows: 2,3-butanedione (≥99.0%), 3-ethyl-2,5-dimethylpyrazine (98%), and malic acid from Macklin Biochemical Technology Corporation (Shanghai, China); 3-hydroxy-2-butanone (98%) and 2,3,5,6-tetramethylpyrazine from Aladdin Industries Corporation (Shanghai, China); and 2,3,5-trimethylpyrazine (>98.0%), 2,3-dimethylpyrazine (>98.0%), 2,4,5-trimethyloxazole (>98.0%), 2,5-dimethylpyrazine (>98.0%), 1-octen-3-ol (>98.0%), guaiacol (>98.0%), and 2-methylbutanoic acid (>98.0%) from TCI Co., Ltd. (Shanghai, China). Propylene glycol (99%) and anhydrous ethanol (≥99.7%) were purchased from Solarbio Science and Technology Co., Ltd. (Beijing, China).

### 2.3. Reverse Transcription-Quantitative Real-Time PCR (RT-qPCR)

Total RNA was extracted using the Column Bacterial Total RNA Extraction and Purification Kit (Sangon Biotech, Shanghai, China), following the manufacturer’s instructions. First-strand cDNA synthesis from total RNA was performed using StarScript III RT Mix with a gDNA Remover (Genstar, Beijing, China). Quantitative real-time PCR (qRT-PCR) was carried out using 2 × RealStar Green Fast Mixture (Genstar, Beijing, China). Expression levels were determined based on Ct values, and the data were normalized using the reference gene 16S rRNA. Primers for both the internal reference and target genes are listed in Table S1. Relative gene expression was normalized using the 2^−ΔΔCT^ method. Each RT-qPCR experiment was performed in triplicate [25].

### 2.4. Construction and Transformation of Homologous Recombinant Knockout Vectors

HLarm stands for the homologous left arm of the target gene, while HRarm represents the homologous right arm. The cm gene refers to chloramphenicol resistance, and erm denotes erythromycin resistance. DEDP is a double-exchange assay primer (Table S1) used to verify successful knockouts. To construct the knockdown vectors, the HLarm and HRarm sequences of *ilvA* and *serA* were PCR-amplified from the *Bacillus subtilis* BJ3-2 genome. The cm gene was amplified from the PHT01cas9-p43 vector. HLarm, cm, and HRarm fragments were sequentially double-digested with EcoRI/*Kpn*I, *Kpn*I/SalI, and SalI/HindIII, respectively. These fragments were then ligated into the pUC18 vector to create the homologous recombinant knockdown vectors pUC18-HLarm(*ilvA*)-cm-HRarm(*ilvA*) and pUC18-HLarm(*serA*)-cm-HRarm(*serA*). For the *serA* and erm knockdown vectors, *serA* was PCR-amplified from the BJ3-2 genome, and erm was amplified from the pMarA plasmid. These fragments were double-digested with *Kpn*I/BamHI and *Pst*I, respectively, and sequentially ligated into the pUC18 vector to generate the homologous recombinant knockdown vector pUC18-*serA*::*Em^r^*. Following construction, the recombinant plasmids were transformed into *Bacillus subtilis* BJ3-2, as described previously [26,27]. The transformants were confirmed by PCR using DEDP primers (Table S1). Genomic DNA from the transformants was extracted using the Bacterial DNA Kit (OMEGA, Woburn, MA, USA) according to the manufacturer’s instructions and sequenced by Tsingke Biotechnology Co., Ltd. (Beijing, China).

### 2.5. Soy Sauce-like Aroma Fermentation Experiment

BJ3-2, BJ3-2*ΔserA*, BJ3-2*ΔilvA*, and BJ3-2*ΔserAΔilvA* were inoculated into 5 mL of liquid LB medium and incubated at 37 °C for 12 h with agitation at 180 rpm. The soybeans were autoclaved at 121 °C for 20 min. A bacterial suspension of the four strains (OD600 = 0.465) was inoculated into sterilized soybeans (the bacterial inoculation was 1% of the soybean medium) and fermented for 72 h at 53 °C.

### 2.6. Sensory Evaluation

The soybeans’ fermentation medium was employed for simulating the fermentation of soy sauce-like aroma [28]. Upon the completion of the fermentation, sensory evaluation was conducted. To ensure the accurate assessment of fermentation characteristics, a panel of 10 trained sensory evaluators participated in quantitative descriptive analysis (QDA). The panelists, consisting of 5 males and 5 females aged 20–30 years, underwent rigorous three-day training to familiarize themselves with the odors associated with soy sauce-like aroma fermentation. Firstly, a descriptive test was conducted to elicit the sensory descriptor. Each sample from the fermentation with soy sauce-like aroma (20 g each) was put into 50 mL Teflon vessels, and each member smelled them to freely record the sensory descriptors. The panel then discussed and reached a unanimous agreement based on the frequency and intensity of the sensory attributes of the soy sauce-like aroma of fermented soybeans. Through the panelists’ discussion and after reaching a unanimous opinion, six aroma attributes were selected for evaluation: soy sauce-like aroma, roasted, smoky, sweet, buttery, and grain aroma. Roasted aroma evokes scents reminiscent of aromas of chocolate and roasted nuts [9]; smoky aroma resembles the fresh scent of wood and grass [9]; buttery aroma is akin to the aroma of cream and yogurt [9]; sweet aroma resembles caramelized popcorn; and grain aroma is similar to the scent of steamed bean grains [9]. Soy sauce-like aroma is the overall aroma of fermented soybeans and is described as a smooth, lingering aroma akin to that found in saucy fermented products [17]. Samples from the fermentation with soy sauce-like aroma were placed in 50 mL odorless, clear PET bottles, each labeled with a random three-digit number and assigned randomly to assessors for scoring. For each odor attribute, the intensity scores ranged from 1 (faint odor) to 5 (strong odor) in increments of 1 [29]. All experiments were conducted in triplicate, and average scores were calculated for each attribute.

### 2.7. Extraction of Volatile Compounds from Fermentations by HS-SPME

We referred to the HS-SPME technique of Peng et al. for the extraction of volatile compounds in fermentation [30]. Volatiles from fermented soybeans were extracted using a 2 cm 50/30 μm DVB/CAR/PDMS extraction head combined with an autosampler. The extraction head was preconditioned at 270 °C for 30 min prior to the experiment to eliminate residual substances. To prepare the sample, 3 g of fermented soybeans were accurately weighed using a 0.001 g electronic balance and placed into a 20 mL headspace vial. A 5 μL aliquot of 180 μg/mL sec-octanol solution was added as the internal standard, and the vial was sealed. The headspace vial was placed in an incubator-equipped autosampler with a vibration frequency set at 250 rpm. After pre-treatment at 50 °C for 10 min, the extraction head was inserted into the headspace vial for sampling. The sample underwent headspace extraction for 30 min, followed by injection into the GC pre-sample port for analysis, which was conducted over a 3 min period.

### 2.8. GC-MS Analysis

The analytical method of GC-MS was evaluated based on the method of Peng et al., with slight modifications [30]. The measurements were conducted using a 7890-5977B GC-MS system equipped with a polar WB-WAX column under specific experimental conditions. Helium served as the carrier gas at a flow rate of 2 mL/min. The column temperature program was initiated at 40 °C and held for 3 min, followed by an increase to 130 °C at a rate of 3 °C/min. It was maintained at 130 °C for 5 min and then ramped at 4 °C/min to 200 °C and held for 5 min. An electron ionization source was employed with an energy of 70 eV, scanning from *m*/*z* 35 to 500 at a rate of 1.8 scans/s. The inlet temperature was set at 250 °C, the ion source temperature was set at 230 °C, and the quadrupole rod temperature was set at 150 °C. The injection was non-split, with a solvent delay of 3 min.

### 2.9. GC–O Analysis

C-O analysis was conducted on an Agilent 7890B-5977B GC-MS equipped with an ODP4 olfactory detection port. The GC effluent was evenly split between the MS and sniffing port at a 1:1 ratio. Helium (99.99%) served as the carrier gas for continuously separating the volatiles in the column, delivered to the sniffing port at a flow rate of 1.5 mL/min. The delivery line temperature was maintained at 250 °C. To ensure comfort during sniffing, moist air was introduced into the sniffing port. Fermented soybean samples simulating sauces were analyzed by three trained and experienced experimental team members (two males and one female), who conducted an olfactory detection of odor descriptors through sniffing. Each sample was evaluated in triplicate. The panelists rated the intensity of the detected odors on a scale from 1 to 5, with higher ratings indicating stronger odors of the compounds detected by sniffing [31].

### 2.10. Qualitative and Quantitative Analyses

The volatile compounds contributing to the soy sauce-like aroma ferment were identified by comparing their mass spectra with those in the NIST 20.0 database and by referencing retention indices (RI) and odor profiles from relevant literature. RI values were calculated using a homologous series of n-alkane standards (C7–C30) under identical chromatographic conditions. The formulas were derived from comprehensive studies on major volatile compounds. Semi-quantitative analysis of volatile compounds was conducted using 5 µL of sec-octanol (180 µg/mL) as an internal standard. Concentrations of aromatic compounds were determined based on peak areas of the volatiles and the known concentration of the internal standard. The relative abundance of each compound was assessed following the methodology described by Zhao et al. [32]. The concentration of aromatic compounds was calculated using the following formula [32]:

$$C_{x}=\frac{A_{x}\times C_{s}\times V_{s}}{A_{s}\times m_{x}}$$

where C*_x_* is the content of compound *x* (ug/g); *A_x_* and *A_s_* are the peak areas of compound *x* and internal standard *s*, respectively; *m_x_* is the mass of the sample (g); and *C_s_* and vs. are the concentration (µg/mL) and volume (mL) of the added internal standard, respectively.

### 2.11. Relative Odor Activity Values (rOAV) Determination

The rOAV for each volatile substance was calculated using the formula rOAV = Ci/OTi, where Ci represents the relative concentration of the compound and OTi denotes its odor threshold in water. The odor thresholds for the aroma-active compounds were sourced from literature references [33].

### 2.12. Adding Experiments

Based on the sensory evaluation and analysis of variance, it is known that S4 is the sample with the strongest soy sauce-like aroma, and S1 is the sample with the weakest soy sauce-like aroma. Sample S1 was used as the addition matrix. Based on the semi-quantitative results, the difference values of substances positively correlated with the soy sauce-like aroma were calculated, and these substances were added to sample S1. The odors were equilibrated for 10 min, and the evaluators rated both S1 and the added sample S1-A for each odor attribute and overall soy sauce-like aroma.

### 2.13. Data Analysis

At least three replications of each experiment and sample analysis were conducted. The GC-MS results were analyzed and processed using Excel 2019, with experimental data expressed as the mean ± SD. The data were statistically analyzed by SPSS software (version 26.0, Inc., Chicago, IL, USA) and Origin 2021 (OriginLab Corporation, Northampton, MA, USA) using a Fisher-LSD to analyze a significant difference between BJ3-2(S1), BJ3-2*ΔilvA*(S2), BJ3-2*ΔserA*(S3), and BJ3-2*ΔserAΔilvA*(S4) at *p* ≤ 0.05. Sensory evaluation results were visualized using radar plots, while combined rOAV and GC-O analyses were depicted using Venn diagrams. Bar, radar, and Venn diagrams were generated using Origin 2021 (OriginLab Corporation, Northampton, MA, USA), and clustered heatmaps were created using TBtools version 2019 (Heatmap Illustrator, Wuhan, China). Principal Component Analysis (PCA) and Orthogonal Partial Least Squares Discriminant Analysis (OPLS-DA) were performed using SIMCA-P 14.1 (Umetrics, Umeå, Sweden). Correlations between sensory attributes and key aroma compounds were analyzed using PLSR in The Unscrambler version 9.7 software (CAMO ASA, Oslo, Norway).

## 3. Results and Discussion

### 3.1. Expression of ilvA and serA in BJ3-2 Incubated at 37 °C and 53 °C

**Figure 1:**
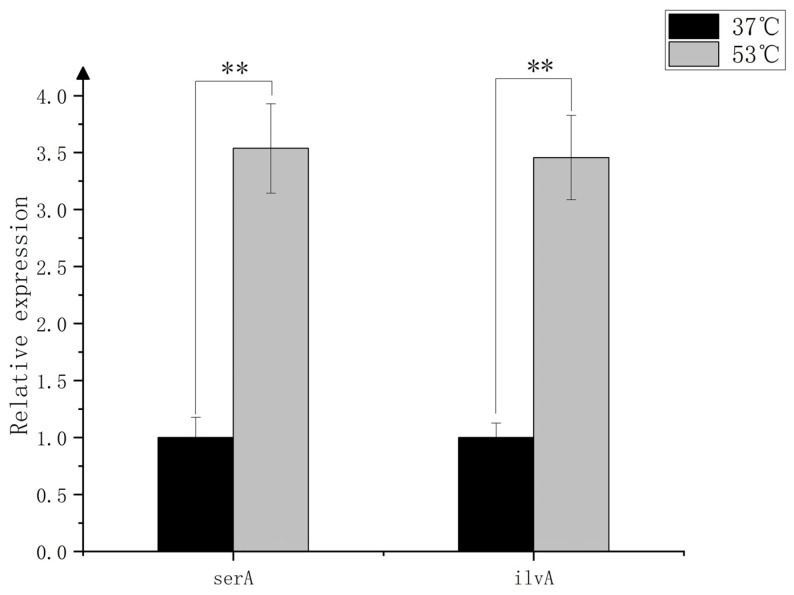
RT-qPCR verified the target gene expression. Using SPSS version 26.0, the data were analyzed using one-way ANOVA and then least significant difference (LSD) to assess the significance of mean differences. ** shows significant differences (*p* < 0.05). The results were calculated by the 2^−ΔΔCT^ method.

The transcriptome, proteome, and metabolome of *Bacillus subtilis* BJ3-2 at 37 °C and 53 °C were previously analyzed [17]. In the current study, we further analyzed and screened the multi-omics data. Screening conditions for differentially expressed genes, proteins, and metabolites were set at |log2FoldChange| ≥ 2, |log2FoldChange| ≥ 1.4, and |log2FoldChange| ≥ 1, respectively, with a significance level of *p* < 0.05. The enriched differentially expressed genes, proteins, and metabolites, along with their associated KEGG metabolic pathways, were jointly analyzed. We found that genes meeting these screening criteria were enriched in the glycine, serine, and threonine metabolic pathways, which have been previously implicated in the literature in relation to the formation of soy sauce-like aroma in *Bacillus subtilis* [21]. Within the glycine, serine, and threonine metabolic pathway, *gbsA*, *ilvA*, and *serA* were highly expressed at 53 °C. The knockdown of *gbsA* also significantly impaired the growth of *Bacillus subtilis* [34]. The |log2FoldChange| values for *ilvA* and *serA* at 53 °C versus 37 °C were 2.00 and 2.16 (Table S2), respectively, identifying *serA* and *ilvA* as candidate genes for further study. We validated the expression of *ilvA* and *serA* in BJ3-2 at both temperatures using RT-qPCR (Table S3), as depicted in Figure 1. The expression of *ilvA* and *serA* was notably higher at 53 °C, consistent with the RNA-seq findings.

### 3.2. Generation of Knockout Strains

Knockout vectors, pUC18-*serA*::*Em^r^* and pUC18-HLarm-cm-HRarm, targeting *serA* and *ilvA*, respectively, were constructed and introduced into *Bacillus subtilis* BJ3-2. Positive clones were confirmed via PCR (Figures S1 and S2). Subsequent sequencing of the positive clones (Figures S3 and S4) revealed the successful replacement of *ilvA* with cm in the BJ3-2 genome using the pUC18-HLarm-cm-HRarm vector and the insertion of erm into *serA* in pUC18-*serA*::*Em^r^*. This confirmed the knockdown of *serA* and *ilvA* in *Bacillus subtilis* BJ3-2, resulting in strains designated as BJ3-2*ΔserA* and BJ3-2*ΔilvA*, respectively. The pUC18-HLarm-cm-HRarm vector was subsequently transformed into BJ3-2*ΔserA*, and positive clones were validated by PCR (Figure S5) and sequenced (Figure S6). These results confirmed the successful knockdown of both *serA* and *ilvA* in *Bacillus subtilis* BJ3-2, generating the double knockdown strain designated as BJ3-2*ΔserAΔilvA*.

### 3.3. Characterisation of Strains

**Figure 2:**
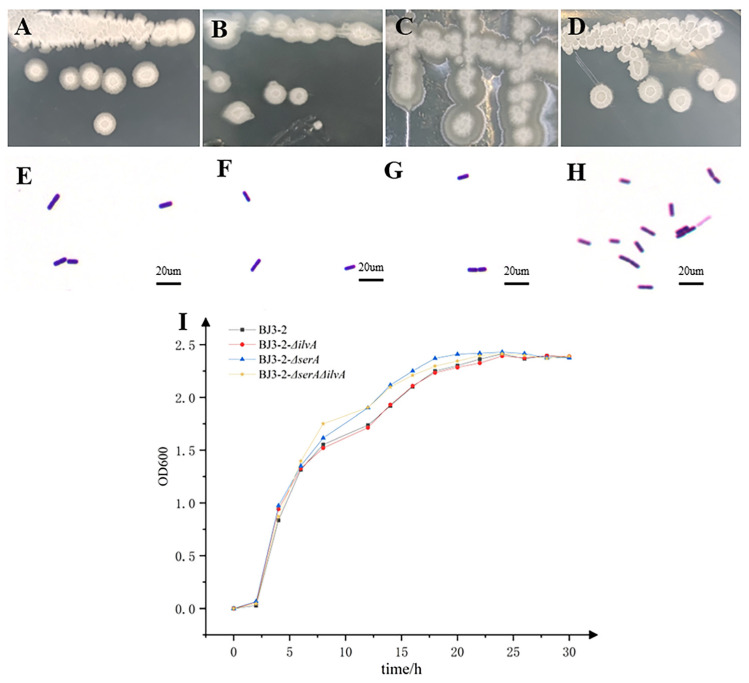
Colony morphology and growth curves. (**A**–**D**) Colony morphology of BJ3-2, BJ3-*2ΔilvA*, BJ3-2*ΔserA*, and BJ3-2*ΔserAΔilvA*; (**E**–**H**) Gram staining of BJ3-2, BJ3-2*ΔilvA*, BJ3-2*ΔserA*, and BJ3-2*ΔserAΔilvA*; (**I**) BJ3-2 Growth curves of BJ3-2*ΔilvA*, BJ3-2*ΔserA*, and BJ3-2*ΔserAΔilvA*.

To assess the growth characteristics of BJ3-2*ΔilvA*, BJ3-2*ΔserA*, and BJ3-2*ΔserAΔilvA*, their appearance on plates showed similar colors and shapes, characterized by rough surfaces and irregular folds (Figure 2A–D). Additionally, Gram staining revealed that BJ3-2*ΔilvA*, BJ3-2*ΔserA*, and BJ3-2*ΔserAΔilvA* exhibited a microscopic morphology of short purple rods, consistent with BJ3-2 (Figure 2E–H). There were no significant differences in morphology or Gram-staining characteristics compared to BJ3-2. Similarly, their growth curves (Figure 2I) demonstrated nearly identical patterns to BJ3-2, indicating comparable growth capacities among BJ3-2*ΔilvA*, BJ3-2*ΔserA*, BJ3-2*ΔserAΔilvA*, and the wild-type strain.

### 3.4. Sensory Evaluation

**Figure 3:**
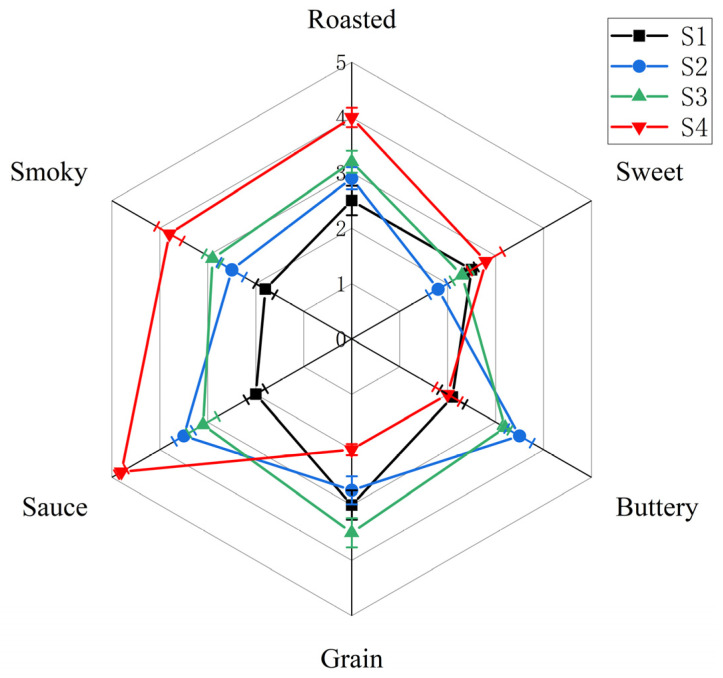
Sensory characteristic of samples S1, S2, S3, and S4 by QDA (S1, S2, S3, and S4 are samples fermented by *Bacillus subtilis* BJ3-2, BJ3-2Δ*ilvA*, BJ3-2*ΔserA*, and BJ3-2*ΔserAΔilvA*, respectively).

Fermented samples (S1, S2, S3, and S4) from four strains (BJ3-2, BJ3-2*ΔilvA*, BJ3-2*ΔserA*, and BJ3-2*ΔserAΔilvA*) were analyzed for their aroma profiles. A QDA analysis based on six sensory attributes was conducted to evaluate the overall aroma profile (Figure 3). In sample S4 of BJ3-2*ΔserAΔilvA* fermentation, both roasted and smoky aromas scored high, suggesting these attributes dominate the soy sauce-like aroma, while sweet, buttery, and grain aromas scored relatively low, indicating lesser influence on the soy sauce-like profile. Differences were observed in the overall soy sauce-like aroma among samples S1, S2, S3, and S4. Samples S2 and S3 exhibited slightly stronger soy sauce-like aromas compared to S1, whereas sample S4 showed a significantly stronger overall soy sauce-like aroma than S1, S2, and S3. In order to analyze whether the differences in soy sauce-like aroma among the four samples were statistically significant, we performed statistical analysis using SPSS. The results of the analysis showed that the sensory differences in soy sauce-like aroma produced by the four samples, S1, S2, S3, and S4, were statistically significant (*p* ≤ 0.05). The soy sauce-like aroma of S2, S3, and S4 was significantly enhanced compared to that of the S1 sample. Meanwhile, the soy sauce-like aroma of S4 was significantly enhanced compared to that of the S2 and S3 samples. Sample S4 specifically displayed intensified roasted and smoky aromas, contributing to its overall more intense soy sauce-like profile. The panelists unanimously agreed that sample S4 had the strongest soy sauce-like aroma. These findings suggest that the knockdown of *ilvA* and *serA* enhances the soy sauce-like aroma during BJ3-2 fermentation. This result prompts further investigation into the specific substances responsible for enhancing this aroma profile.

### 3.5. Qualification and Quantification of Volatile Compounds

SPME and GC-MS detected the volatiles of soy sauce-like aroma fermentation samples fermented by different knockout strains. An internal standard, sec-octanol, was added for quantification, with the results shown in Table S4. A total of 99 compounds were identified and categorized into pyrazines (13), ketones (19), acids (6), alcohols (22), aldehydes (16), phenols (3), and others. Compared to S1 (10338.15 μg/kg), a sample of soy sauce-like aroma fermentation using *Bacillus subtilis* BJ3-2, other samples fermented by knockout strains (S2: 11061.10 μg/kg, S3: 11048.72 μg/kg, and S4: 12366.8530 μg/kg) showed an overall increase in the total amount. Possibly due to the knockout of *serA* and *ilvA*, altering metabolic pathways involving serine, glycine, and threonine thereby increased pyrazines and other compounds, leading to a higher total amount.

**Figure 4:**
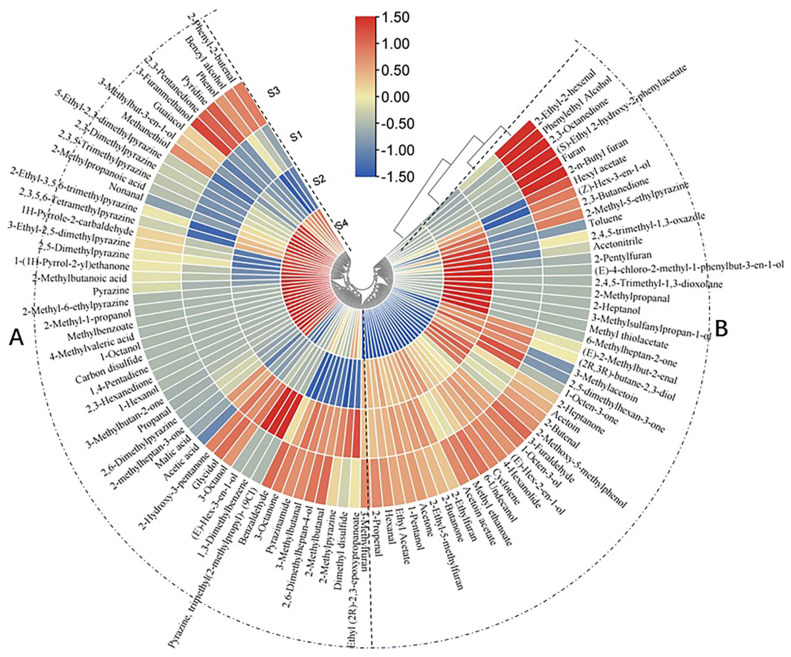
Hierarchical clustering and thermogram visualization of volatile compounds for four samples. High concentrations are represented in red, while low concentrations are represented in blue. A and B: According to the cluster analysis of the heat map, all the compounds detected in the four samples were clustered into two groups, group A and group B respectively.

To facilitate a clearer comparison among the four samples of soy sauce-like fermentations, volatile compounds were analyzed using a clustered heat map (Figure 4). This heat map illustrates the average content distribution of each volatile through color shades, employing hierarchical cluster analysis (HCA). The color intensity ranges from maximum (red) to minimum (blue), indicating consistency from high to low volatile contents [35]. The HCA results categorized the fermentation samples into two main groups: one comprising S1, S2, and S3 and the other consisting of S4, aligning with sensory evaluation outcomes. This observation suggests that the simultaneous knockdown of *serA* and *ilvA* significantly impacts the soy sauce-like aroma.

In addition, volatile compounds were classified into two main groups according to the tree diagram. Group A comprised 60 compounds, encompassing various classes such as acids and pyrazines. Tetramethylpyrazine and 2,3,5-trimethylpyrazine were notably higher in Group A, potentially contributing significantly to the soy sauce-like aroma. Previous studies indicated that pyrazines such as tetramethylpyrazine and 2,3,5-trimethylpyrazine impart predominantly nutty, cocoa, and chocolate aromas, thereby influencing the roasted composition in the soy sauce-like aroma of the samples [36]. These compounds are considered crucial flavor contributors to the soy sauce-like aroma profile of Moutai, a type of sauce baijiu [7]. Guaiacol, known for its smoky and herbaceous odor, was predominantly detected in foods with a soy sauce-like aroma [7]. Samples from the experimental group exhibited significantly higher levels of guaiacol, correlating with a notable enhancement of the soy sauce-like aroma. Compared to S1 acids, S4 exhibited a 25.85% increase. Research has demonstrated that acidic compounds are particularly conducive to enhancing the soy sauce-like aroma, playing a pivotal role in enriching the overall aromatic profile [2]. This finding underscores their importance in enhancing the richness of the soy sauce-like aroma in S4 samples.

Group B comprised 47 compounds, predominantly ketones and others. Compared to S1, ketones in S2 increased by 18.92% and in S3 by 12.56%. It has been shown that the increase in ketones can enrich the soy sauce-like aroma [2]. Notably, 2,4,5-trimethyloxazole may be closely related to the formation of soy sauce-like aroma [7]. In the present experiments, 2,4,5-trimethyloxazole was also detected, with relatively higher levels in S2 and S3 compared to S1 samples. These findings suggest that the increased presence of 2,4,5-trimethyloxazole and ketones may significantly contribute to enhancing the soy sauce-like aroma in S2 and S3 samples compared to S1.

### 3.6. Characteristic Flavor Substances of Soy Sauce-like Aroma by rOAV and GC-O

Numerous studies have demonstrated that many volatile compounds in food matrices may not significantly contribute to the aroma profile, with only a small fraction being involved in aroma perception [37]. Odor Specific Magnitude Estimation (OSME) is an effective method for evaluating compounds responsible for aroma perception based on Aroma Intensity (AI) [38], where compounds with stronger aromas exhibit higher AI values [39]. The contribution of aroma compounds to the soy sauce-like aroma depends not only on their concentration but also on their odor thresholds and interactions [35]. To further assess the contribution of aroma-active compounds to the aroma characteristics of sauces, their rOAV are often used. rOAV is the ratio of the concentration of volatile compounds to their odor thresholds, with compounds having rOAV ≥ 1 considered significant contributors to the aroma profile [40]. Therefore, identifying characteristic aroma-active compounds among the 99 compounds in the four samples is crucial for understanding soy sauce-like aroma formation, often analyzed through a combination of rOAV and GC-O.

Nineteen aroma actives were identified through rOAV analysis (Table S5), with 15, 15, 16, and 18 compounds in S1 to S4 having rOAV values above 1, respectively. Fifteen of these compounds exhibited rOAV > 1 across all four samples. Among them, guaiacol (rOAV 320.4–693.4), 2,3,5-trimethylpyrazine (rOAV 123.6–141.6), 2,3-butanedione (rOAV 364.3–449.7), and 2,5-dimethylpyrazine (rOAV 41.7–71.3) showed particularly high rOAV values, indicating their significant contribution to the soy sauce-like aroma. Additionally, 1-octen-3-ol (rOAV 28.9–36.1), 2,4,5-trimethyloxazole (rOAV 10.9–26.3), dimethyl disulfide (rOAV 24.8–29.7), 2,3,5,6-tetramethylpyrazine (rOAV 6.0–9.54), 3-hydroxy-2-butanone (rOAV 3.09–5.24), 3-ethyl-2,5-dimethylpyrazine (rOAV 1.8–3.2), malic acid (rOAV 0.96–2.71), 3-methylbutyric acid (rOAV 0.97–1.36), 2-methylbutyraldehyde (rOAV 9.4–13.4), 2-ethylfuran (rOAV 6.6–7.4), and 2-n-pentylfuran (rOAV 2.7–5.3) exhibited relatively lower rOAV values but still contributed significantly to the overall aroma.

Only a small fraction of compounds in the volatile components of food matrices have an aroma effect. GC-O is commonly used to assess the odor intensity of compounds detectable by experimenters [41]. GC-O intensity is rated by experimenters based on the perceived level of odor intensity of the compound, with aromatic compounds detected by more than two experimenters being recorded. As detailed in Table S6, a total of 17 volatile active compounds were identified from the four samples through GC-O experiments, including eight pyrazines, two alcohols, three acids, two ketones, and one each of oxazoles and phenols. These volatiles are presumed to be key flavor substances in fermented sauces. Specifically, 2,3,5-trimethylpyrazine, 2,3-butanedione, guaiacol, 2,5-dimethylpyrazine, malic acid, 2-methylbutanoic acid, and 3-hydroxy-2-butanone exhibited high GC-O intensities (≥3) across samples, indicating their significant contribution to the sauce’s aroma profile.

**Figure 5:**
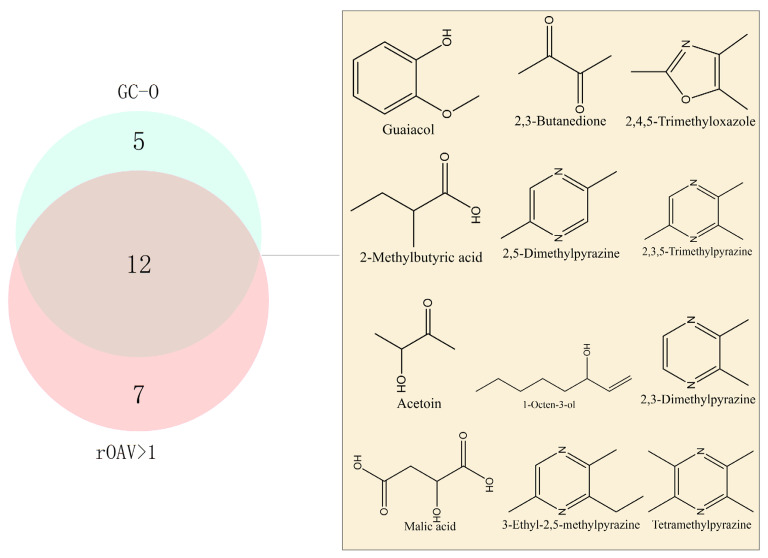
Analysis of aroma-active compounds in soy sauce by rOAV and GC-O.

The results of rOAV analysis and GC-O detection were further analyzed using a Venn diagram (Figure 5). This combined analysis revealed 12 characteristic flavor compounds detected across all four samples, including 2,3,5-trimethylpyrazine, 2,5-dimethylpyrazine, 2,3,5,6-tetramethylpyrazine, 2,3-dimethylpyrazine, 3-ethyl-2,5-dimethylpyrazine, guaiacol, malic acid, 2-methylbutanoic acid, 2,3-butanedione, 3-hydroxy-2-butanone, 1-octen-3-ol, and 2,4,5-trimethyloxazole. These compounds are identified as key aroma contributors influencing the soy sauce-like aroma.

They were categorized into four groups based on aroma characteristics, detailed in Table 1. Group 1 (2,3,5-Trimethylpyrazine, 2,5-Dimethylpyrazine, 2,3,5,6-tetramethylpyrazine, 2,3-Dimethylpyrazine, and 3-Ethyl-2,5-Dimethylpyrazine) exhibits chocolatey, nutty, and baked-potato aromas, predominantly contributing roasted notes. Group 2 (2,3-butanedione, 3-hydroxy-2-butanone, and malic acid) presents pleasant aromas reminiscent of yogurt, buttery, and burnt sugar. Group 3 (guaiacol and 2,4,5-trimethyloxazole) imparts grassy, woody, and cucumber aromas, offering a refreshing scent. Group 4 (2-methylbutyric acid and 1-octen-3-ol) is characterized by a slightly pungent odor resembling sweat and peas. These key aroma compounds form the foundation of the soy sauce-like aroma. Additionally, four compounds were exclusively detected by GC-O, and seven compounds showed rOAV values > 1 only. These aromatic compounds likely play a crucial role in harmonizing the overall soy sauce-like aroma.

### 3.7. PCA and OPLS-DA Analyses

**Figure 6:**
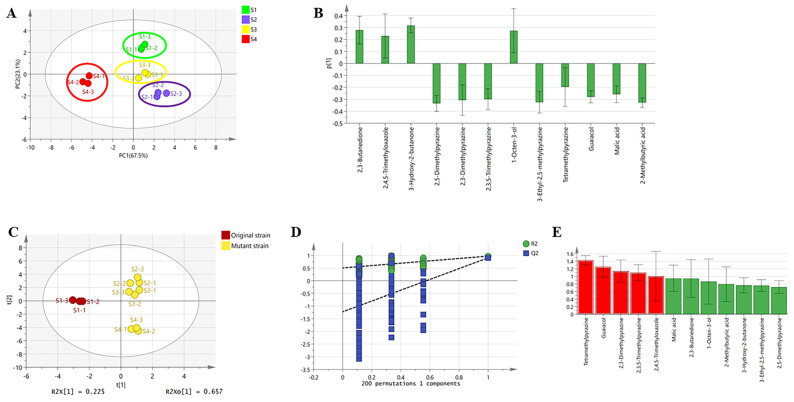
PCA and OPLS-DA analyses were performed on four samples S1–S4 with different sauce intensities. (**A**) PCA score plots; (**B**) PCA loading column plot; (**C**) OPLS-DA score plots; (**D**) 200 permutation test cross-validation plots; and (**E**) VIP values, where compounds with a VIP > 1 are highlighted in red, indicating their significance.

Based on the content of key flavor compounds with rOAV > 1 or detected by GC-O, and to better visualize the variability between samples with different degrees of sauce intensity, multivariate analysis (PCA and OPLS-DA) was conducted in this study. PCA is an unsupervised data analysis method for visualizing differences in complex information between samples without prior knowledge of the dataset [42]. PCA analysis of the 12 aforementioned flavor compounds in each sample revealed that the two principal components, PC1 and PC2, accounted for over 80% of the total variance, indicating that the model effectively explains sample variability. As depicted in Figure 6A, the 12 samples from the 4-sample group formed four distinct clusters. Sample S1 was located at the positive end of PC2, while S2, S3, and S4 were located at the negative end of PC2, clearly separated from each other. At the same time, sample S4, characterized by the strongest soy sauce-like aroma, was positioned at the negative end of PC1, while S1, S2, and S3 were positioned at the positive end and were separable from each other along PC2. And we also analyzed the PC1 loadings plot. By analyzing these loadings, it is possible to reveal the correlations between the variables and their influence on the formation of the principal component [43]. In Figure 6B, it can be observed that 2,3-butanedione, 2,4,5-trimethyloxazole, 3-hydroxy-2-butanone, and 1-octen-3-ol are positive signals, and 2,3,5-trimethylpyrazine, 2,3-dimethylpyrazine, 3-ethyl-2,5-methylpyrazine, tetramethylpyrazine, 2,5-dimethylpyrazine, malic acid, guaiacol, and 2-methylbutanoic acid are negative signals. Whereas the height of the column reflects the degree of contribution to the principal components, in Figure 6B, it can be observed that pyrazines and guaiacol contribute to the principal components to a greater extent. Notably, in the PCA score plot, the distance between S4 and S1, S2, and S3 was significant, consistent with sensory evaluation results indicating a pronounced difference between S4 and the other three samples. This suggests that the simultaneous knockdown of *serA* and *ilvA* significantly influences the soy sauce-like aroma in fermentation, enhancing the overall soy sauce-like aroma, as observed in sensory experiments.

Based on PCA analysis, to further investigate differences between samples exhibiting varying levels of soy sauce-like aroma intensity, subsequently, OPLS-DA analysis was performed for 12 aroma actives. OPLS-DA, a discriminant analysis method, maximizes the interpretation of between-group differences using sample grouping information [44]. The samples were classified based on fermentation strains: one group included sample S1 fermented by the original strain BJ3-2, and the other comprised samples S2, S3, and S4 fermented by mutant strains. These groups were analyzed using OPLS-DA. Figure 6C depicts the OPLS-DA scores plot showing two well-separated clusters (R2 Y = 0.975), indicating high predictive efficacy (Q2 = 0.907). In order to assess whether the OPLS-DA model is overfitting, we performed alignment tests (*n* = 200) on the OPLS-DA model separately, as shown in Figure 6D. Figure 6D illustrates that RY2 exceeds QY2, affirming the good predictability and reliability of the OPLS-DA model without overfitting, as verified by 200 permutation tests. At the same time, we performed a CV-ANOVA test on the model of OPLS-DA. In the CV-ANOVA test result, the *p*-value should be as low as possible (less than 0.05) and the F-value should be as high as possible (greater than 3.85). The F-value of the CV-ANOVA test result is 9.4, which is greater than 3.85, and the *p*-value of the CV-ANOVA test result is 0.006, which is less than 0.05; this also indicates that there is no overfitting of the OPLS-DA analytical model. VIP was used to identify the most significant variables, and it is generally accepted that variables with a VIP greater than 1 are considered to have a significant impact on the discrimination of samples. Figure 6E demonstrates the VIP values for varying compounds. Variables with VIP values exceeding 1, including 2,3,5,6-tetramethylpyrazine, 2,3,5-trimethylpyrazine, guaiacol, 2,3-dimethylpyrazine, and 2,4,5-trimethyloxazole (VIP > 1), are significant for sample discrimination. The detection of these five differential flavor volatiles can serve as markers for distinguishing enhanced soy sauce-like aroma intensity.

### 3.8. Correlation between Key Aroma Compounds and Sensory Attributes

**Figure 7:**
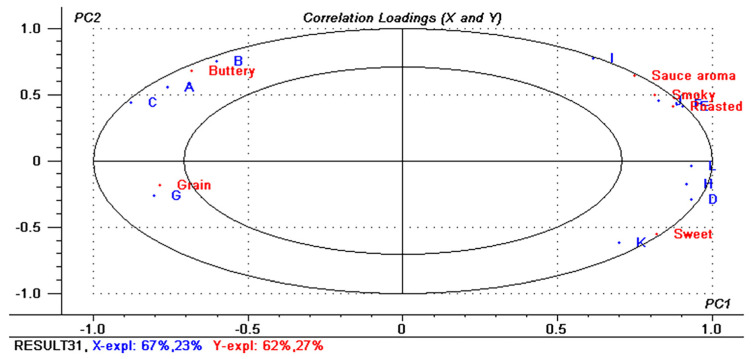
An overview of the variation found in the mean data from the partial least-squares regression (PLSR) correlation loading plot of sensory attributes and key aroma compounds. Ellipses represent r^2^ = 0.5 and 1.0 ((A) 2,3-butanedione; (B) 2,3,5-trimethylpyrazine; (C) 3-hydroxy-2-butanone; (D) 2,5-dimethylpyrazine; (E) 2,3-dimethylpyrazine; (F) 2,3,5-trimethylpyrazine; (G) 1-octen-3-ol; (H) 3-ethyl-2,5-dimethylpyrazine; (I) 2,3,5,6-tetramethylpyrazine; (J) guaiacol; (K) malic acid).

Partial Least Squares Regression (PLSR) was utilized to establish correlations between identified key aroma compounds and sensory attributes. PLSR comprised two principal components explaining 90% of the variance in X (12 key aroma compounds) and 89% in Y (six sensory attributes) (Figure 7). The inner and outer ellipses represent 50% and 100% of the explained variance, respectively, intersecting at the variances’ location, indicating a well-explained PLSR model. The soy sauce-like aroma exhibited positive correlations with smoky and roasted attributes. These findings align with sensory evaluations (Figure 1) and GC-O analysis. Pyrazines such as 2,3,5-trimethylpyrazine, 2,5-dimethylpyrazine, 2,3-dimethylpyrazine, 3-ethyl-2,5-dimethylpyrazine, and 2,3,5,6-tetramethylpyrazine showed strong correlations with roasted aromas; guaiacol was closely associated with smoky aromas; malic acid correlated strongly with sweet aromas; and 3-hydroxy-2-butanone and 2,3-butanedione were highly correlated with creamy aromas. These aroma-active compounds, identified as key aroma contributors in soy sauce-like foods, are consistent with findings in previous studies [2,3,7,11].

**Figure 8:**
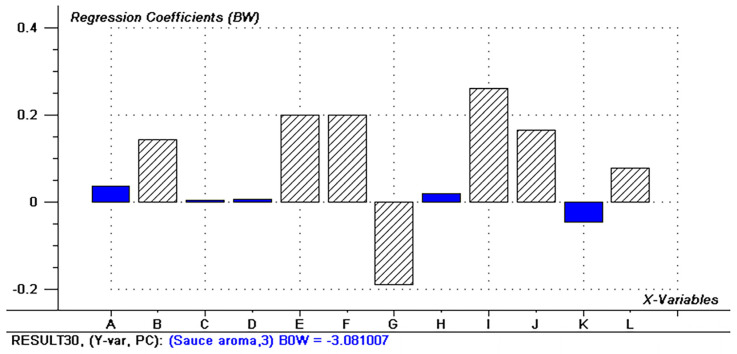
Regression coefficients and significance indicators of sensory attributes derived from PLS1: sauce aroma.

To further explore which volatile compounds significantly contributed to sensory attributes, PLS1 regression analyses were conducted. Additionally, significant attribute variables were examined by calculating estimated regression coefficients using the jack-knife uncertainty test [45]. Figure 8 illustrates that 2,3,5-trimethylpyrazine, 2,5-dimethylpyrazine, 2,3-dimethylpyrazine, 3-ethyl-2,5-dimethylpyrazine, 2,3,5,6-tetramethylpyrazine, guaiacol, 3-hydroxy-2-butanone, 2,3-butanedione, 2,4,5-trimethyloxazole, and 2-methylbutyric acid were positively correlated with the soy sauce-like aroma. Notably, pyrazines and guaiacol were the most influential contributors to the soy sauce-like aroma, while ketones and acids also made significant contributions.

Combining the above data and results reveals that pyrazines, phenolic compounds, and ketones notably enhance the soy sauce-like aroma. This aroma profile comprises a complex system of baked, smoky, sweet, creamy, and cereal notes that harmoniously interact. Based on these characteristics and changes in the compound content during sensory evaluation, it is evident that pyrazines (2,3,5,6-tetramethylpyrazine, 2,3,5-trimethylpyrazine, 2,5-dimethylpyrazine, 2,3-dimethylpyrazine, 3-ethyl-2,5-dimethylpyrazine, and 2,4,5-trimethyloxazole) and phenols (guaiacol) make substantial contributions to the soy sauce-like aroma. Ketones (2,3-butanedione, 3-hydroxy-2-butanone), alcohols, and acids also contribute, forming a complex soy sauce-like aroma system.

### 3.9. Changes in Key Aroma Compounds of the Soy Sauce-like Aroma in Fermented Samples between BJ3-2, BJ3-2ΔilvA, BJ3-2ΔserA, and BJ3-2ΔserAΔilvA

**Figure 9:**
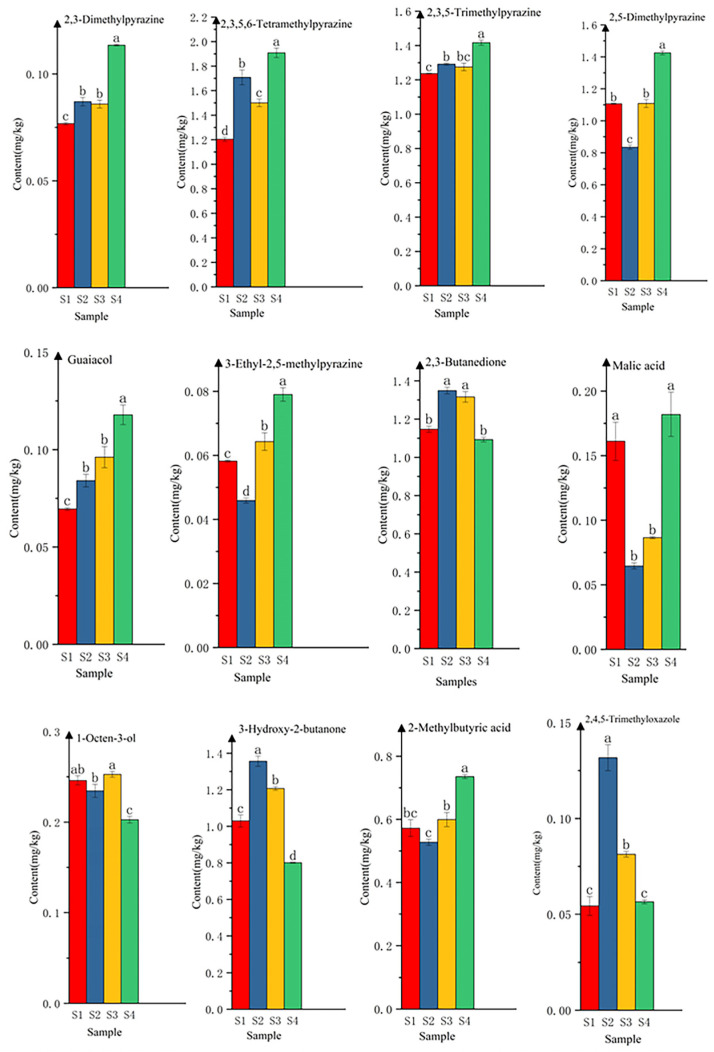
Changes in the content of key aroma compounds among the four fermented soybean samples with BJ3-2, BJ3-2*ΔilvA*, BJ3-2*ΔserA*, and BJ3-2*ΔilvAΔserA*. Significant differences between groups are indicated by letters a, b, d, c.

A total of 12 key aroma compounds from four samples were analyzed for significance of differences. The results of the analysis showed statistically significant (*p* ≤ 0.05) differences in the content of the 12 key aroma compounds among the four fermented samples (S1, S2, S3, and S4) produced from BJ3-2, BJ3-2*ΔilvA*, BJ3-2*ΔserA*, and BJ3-2*ΔserAΔilvA* fermented soybeans. As shown in Figure 9, the contents of tetramethylpyrazine, 2,3,5-trimethylpyrazine, and 2,3-dimethylpyrazine were significantly increased in the S2, S3, and S4 samples added with BJ3-2*ΔilvA*, BJ3-2*ΔserA,* and BJ3-2*ΔserAΔilvA*, as compared to the S1 samples fermented from BJ3-2. Among them, the fermented samples of BJ3-2*ΔserAΔilvA* had the highest content of tetramethylpyrazine, 2,3,5-trimethylpyrazine, 2,3-dimethylpyrazine, and other pyrazines. The highest pyrazines content in the BJ3-2*ΔserAΔilvA* fermented samples also explains why it has the highest intensity of “roasted” properties (Figure 3). A significant increase in guaiacol was also seen in the fermentation samples of BJ3-2*ΔilvA*, BJ3-2*ΔserA*, and BJ3-2*ΔilvAΔserA*, which is in agreement with the results of the QDA of the “smoky” enhancement in S2, S3, and S4 (Figure 3). On the other hand, 1-octen-3-ol and 3-hydroxy-2-butanone were significantly reduced in BJ3-2*ΔilvAΔserA*, which is in line with the QDA results of sample S4, with reduced “grain” and “buttery“ aromas (Figure 3). These results also show consistency with the PLSR results (Figure 7) as well. Several other compounds also showed some variation in content between the four samples.

In addition, KEGG pathway analysis showed that *ilvA* (encoding threonine dehydratase) and *serA* (encoding D-3-phosphoglycerate dehydrogenase) participate in metabolic pathways that are glycine-, serine-, and threonine-metabolizing routes [18,19]. This suggests that the knockout of *ilvA* and *serA* may affect the production of aroma compounds by influencing this metabolic pathway, resulting in altered sensory attributes of BJ3-2 and its mutants that produce soy sauce-like aroma. The effects of the knockdown of *ilvA* and *serA* on the formation of soy sauce-like aroma compounds can therefore be further analyzed based on the results of KEGG pathway and differential analysis. First, the knockout of *serA* leads to the deregulation of the feedback inhibition of serine [46], which may lead to an increase in the serine and 3P-glycerate content, which in turn leads to an increase in the pyruvate content. And the increase in the pyruvate content may lead to an increase in the 2,3-butanedione and 3-hydroxy-2-butanone content. It has been documented that when oxygen is present, 2,3-butanedione is destabilized by the reduction to 3-hydroxy-2-butanone [47], which potentially contributes to the increase in the tetramethylpyrazine content. If *ilvA* were knocked out, there would theoretically be a relative increase in the content of L-threonine, which could lead to an increase in the content of aminoacetone. Aminoacetone is a precursor substance for the synthesis of dimethylpyrazine and trimethylpyrazine [21]. In turn, aminopyruvic acid was generated by amine oxidase, aldo-keto transferase, and D-lactate dehydrogenase [21], and NH4+ was also generated in this process, which resulted in an increase in trimethylpyrazine and tetramethylpyrazine levels. Theoretically, the content of 2,5-dimethylpyrazine should have also increased, but the results showed a decrease, presumably due to the preferential synthesis of pyruvic acid by aminopyruvic acetone without the spontaneous reaction to produce 2,5-dimethylpyrazine. The simultaneous knockout of *ilvA* and *serA* may had led to an increase in the pyruvate and aminopyruvate content, which may have then led to an increase in the dimethylpyrazine and trimethylpyrazine content. At the same time, the metabolism of amino acids, such as a threonine and a serine, provides large amounts of NH4+ [20], which may lead to an increase in the tetramethylpyrazine content. The BJ3-2*ΔserAΔilvA* fermentation samples exhibited reduced levels of 3-hydroxy-2-butanone and 2,3-butanedione, which was hypothesized to be due to the large amount of 3-hydroxy-2-butanone and 2,3-butanedione involved in the massive synthesis of 2,3,5-trimethylpyrazine and tetramethylpyrazine leading to the reduction in their levels. From the above analysis, it can be suggested that *serA* and *ilvA* may be the main factors affecting the formation of soy sauce-like aroma by influencing the production of key aroma substances such as pyrazines. However, the roles and specific regulatory mechanisms of *ilvA* and *serA* in the production of the soy sauce-like aroma still need to be further investigated.

### 3.10. Adding Experiments

**Figure 10:**
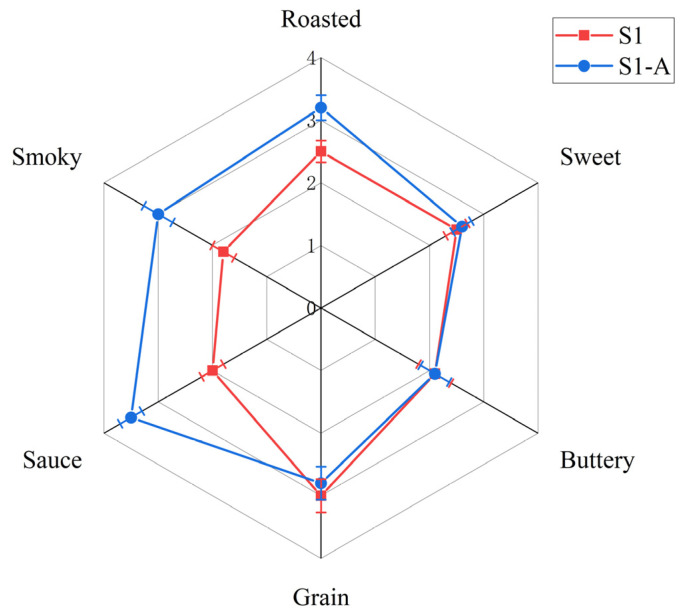
Aroma characteristics of S1 and additive models. (The blue line is the aroma profile of the S1 sample, and the red line is the aroma profile of the addition model).

To verify whether 2,3,5-trimethylpyrazine, 2,4,5-trimethyloxazole, 2,5-dimethylpyrazine, 2,3,5,6-tetramethylpyrazine, 2,3-dimethylpyrazine, 3-ethyl-2,5-methylpyrazine, guaiacol, and 2-methylbutyric acid were positively correlated with soy sauce-like aroma, based on the results of the semi-quantitative study, these compounds were added to the S1 samples. After 10 min of odor equilibration, sensory evaluation was performed on the added sample (S1-A) and the original sample (S1). As shown in Figure 10, the blue line is the aroma profile of the S1 sample, and the red line is the aroma profile of the addition model. Compared with the S1 sample, the overall aroma profile of the additive model showed a significant increase in the intensity of roasted, smoky, and soy sauce-like aromas, which was closer to the profile of the S4 sample. This result indicates that the addition of these seven substances is indeed positively correlated with soy sauce-like aroma. The data suggest that the addition model can well represent that the saucy aroma is enhanced by the addition of these substances, but further considerations are needed to fully establish this. In summary, the soy sauce-like aroma of S1-A was significantly enhanced. The overall sensory evaluation scores suggest that these substances have an important contribution to the soy sauce-like aroma and can enhance the soy sauce aroma.

## 4. Conclusions

In this study, we analyzed the soy sauce-like aroma characteristics and major aroma compounds of fermented samples of Bacillus subtilis BJ3-2 and its knockout mutant at 53 °C by using QDA, GC-O-MS, rOAV, PCA, OPLS-DA, PLSR, and an aroma addition experiment. The QDA results showed that the overall aroma of the soy sauce-like aroma in the fermented samples consisted of “roasted”, “smoky”, “buttery”, “grain”, and “sweet” aromas. In addition, the knockdown of the candidate genes for the soy sauce-like aroma (*serA* and *ilvA*) did not significantly affect the morphology and growth of BJ3-2 but had a greater effect on the aroma profile of soy sauce-like aroma in the fermented samples of BJ3-2. In summary, *serA* and *ilvA* genes of the *B. subtilis* BJ3-2 strain, unfavorable for producing soy sauce-like aroma at 53 °C, were knocked out via homologous recombination. This led to the generation of the mutant strains BJ3-2-*ΔserA*, BJ3-2-*ΔilvA*, and BJ3-2-*ΔserAΔilvA*. These mutant strains, along with the wild strain, were inoculated into the soybean fermentation medium. Sensory evaluations indicated that fermentation by the mutant strains resulted in a stronger soy sauce-like aroma, with BJ3-2-*ΔserAΔilvA* (Sample S4) producing the most pronounced aroma, significantly enhanced compared to that of BJ3-2 (Sample S1). Knockouts of *serA* and *ilvA* result in significant increases and decreases in the levels of key aroma compounds such as pyrazines. Combining sensory evaluations with analyses of differential volatiles and PLSR revealed that pyrazines (2,3,5,6-tetramethylpyrazine, 2,3,5-trimethylpyrazine, 2,5-dimethylpyrazine, 2,3-dimethylpyrazine, and 3-ethyl-2,5-dimethylpyrazine) and phenols (guaiacol) were the primary contributors to the soy sauce-like aroma. These compounds, along with 2,3-butanedione, 2,4,5-trimethyloxazole, 1-octen-3-ol, 2-methylbutyric acid, and other ketones, alcohols, and acids, collectively formed a complex soy sauce-like aroma system. The results of this study provide new insights for analyzing the soy sauce-like aroma of fermented soybeans produced by Bacillus subtilis BJ3-2, which provides a theoretical basis for the quality control and aroma improvement of the soy sauce aroma in fermented foods.

## Figures and Tables

**Table 1:** Odor descriptions of the main aroma compounds in the four samples.

No.	Compounds	Odor Description ^a^	Related Sample ^b^
Group 1			
	2,3,5-trimethylpyrazine	roasted peanut, cocoa	all
	2,5-dimethylpyrazine	woody grass medical	all
	2,3,5,6-tetramethylpyrazine	chocolate, nutty	all
	3-ethyl-2,5-methylpyrazine	roasted potato	all
	2,3-dimethylpyrazine	nutty, coffee	S4
Group 2			
	2.3-butanedione	strong butter, yoghurt	all
	3-hydroxy-2-butanone	buttery, creamy	all
	malic acid	caramel, popcorn	all
Group 3			
	guaiacol	smoky, woody	all
	2,4,5-trimethyloxazole	earthy, cucumbers	all
Group 4			
	2-methylbutyric acid	stinky feet, cheese	all
	1-octen-3-ol	mushroom.	all

^a^: Description of the odor detected by the GC-O sniffer port. ^b^ indicates the sample where the compound was detected by GC-O and rOAV > 1.

## Data Availability

The original contributions presented in the study are included in the article, further inquiries can be directed to the corresponding author.

## Supplementary Materials

The following supporting information can be downloaded at: https://www.mdpi.com/article/10.3390/foods13172731/s1, Table S1: Primers used in this study; Table S2: Transcriptome data at 37 °C and 53 °C; Table S3: Reverse transcription-quantitative real-time PCR at 37 °C and 53 °C; Table S4: Relative content (ug/kg) of volatile compounds in FSs by HS-SPME-GC-O-MS (µg/kg); Table S5: rOAVs of the odor-active substances (rOAV > 1) in samples; Table S6: Odor intensity of the odorants in four samples identified using GC-O; Figure S1: *ilvA* homologous recombination knockout vector construction and transformation of the vector; Figure S2: *serA* homologous recombination knockout vector construction and transformation of the vector; Figure S3: Sequencing of BJ3-2*ΔilvA*; Figure S4: Sequencing of BJ3-2*ΔserA*; Figure S5: PCR validation of BJ3-2*ΔserAΔilvA*; Figure S6: Sequencing of BJ3-2*ΔserAΔilvA*.
