# Supplementary material for: Characterization of Key Aroma Compounds of Soy Sauce-like Aroma Produced in Ferment of Soybeans by Bacillus subtilis BJ3-2
Source: Foods. 2024 Aug 28;13(17):2731. doi: 10.3390/foods13172731 (PMC11395551; doi:10.3390/foods13172731)
Supplement: Supplementary file 1 [file foods-13-02731-s001.zip › Supplementary file S1.pdf]

Supplementary tables

Table S1. Primers used in this study

Name	Table S1. Primers	Enzyme site	Amplic on size
ilvA HLarm	F: CCCAAGCTTAAGCACCTGAGTTTCCGTTA<br>R: ACGCGTCGACGTACAGATTCCTTTCTTGTTTTA	*Eco*R I<br>*Kpn* I	522bp
Cm	F: CGGGGTACCATAGTGACTGGCGATGCTG<br>R: ACGCGTCGACTTAAGTTATTGGTATGACTGGTT	*Kpn* I<br>*Sal* I	1083 bp
ilvA HRarm	F: CCGGAATTCCTAACCTGTCCTTACTGTCCCA<br>R: CGGGGTACCTTTTTTTCGCAACCGCGCAAAATG	*Sal* I<br>*Hin*d III	446 bp
ilvA DEDP	F: ATTCTGGCAGTGTGAGCGTTA<br>R: CAACACTAAATGATACGATGCG		2591 bp
serA HLarm	F: CCCGAGCTCTTTCACTTCACTCTCTCCTT<br>R: CGGGGTACCCGTTAGATTTCCTCCTAA	*Sac* I<br>*Kpn* I	502 bp
Cm	F: CGGGGTACCATAGTGACTGGCGATGCTG<br>R: CGCGGATCCTTAAGTTATTGGTATGACTGGTT	*Kpn* I<br>*Bam*H I	1083 bp
serA HRarm	F: CGCGGATCCTCAAAAAAACTCAAGCTA<br>R: ACGCGTCGACAGACCGTCCCATTATTACA	*Bam*H I<br>*Sal* I	223 bp
serA DEDP1	F: ATCAAAATGGTCATCGCTG<br>R: GAAGCCTGGAAATAGACGAA		2489 bp
serA	F: ACGCGTCGACCGGTTTACAGCCACTT<br>R: ACATGCATGCCAGGGACATTTGTTAGTTC	*Sph* I<br>*Sal* I	1505 bp
Erm	F: AACTGCAGAATTAAGTCGTTAAACCGTGTGC<br>R: AACTGCAGAGGTGTCACAAGACACTCTTTT	*Pst* I<br>*Pst* I	1206 bp
serA DEDP2	F: TTTCACTTCACTCTCTCCTT<br>R: AGACCGTCCCATTATTACA		3509 bp
*serA*RT-qPCR	F: TGCCAAACTTGAAACATCG<br>R: TAAAGGTGCTTCGGTCTCC		203 bp
ilvA RT-qPCR	F: TCCGCCTCACAGCACTCTA<br>R: ATGCCGTCTACTACACCTA		131 bp
16S RT-qPCR	F: ACTCCTACGGGAGGCAGCAG<br>R ATTACCGCGGCTGCTGG		197 bp

Table S2. Transcriptome data at 37℃ and 53℃

	CT53_1	CT53_2	CT53_3	AT37_1	AT37_2	AT37_3	log_2FC（BJ3-2 53℃/37℃）
serA	881.91	982.12	1133.54	222.02	193.67	255.01	2.16
ilvA	550.84	783.55	774.66	208.35	139.05	178.90	2.00

Table S3. Reverse transcription-quantitative real-time PCR at 37°C and 53°C

	37°C-$2^{-\Delta\Delta CT}$	53°C-$2^{-\Delta\Delta CT}$	log_2FC（BJ3-2 53°C/37°C）
serA	1.34	11.52	3.10
	0.85	12.43	3.86
	0.86	10.90	3.65
sdaAA	1.22	9.94	3.30
	0.88	11.03	3.65
	0.94	12.07	3.69

Table S4. Relative content(ug/kg) of volatile compounds in four samples by HS-SPME-GC-O-MS (μg/kg)

No.	Aroma Compounds	CAS	RI[a]	RI[b]	Compound Content (μg /kg)				Identification method[c]
					BJ3-2	BJ3-2Δ*ilvA*	BJ3-2Δ*serA*	BJ3-2Δ*serA*Δ*ilvA*	
A	**Pyrazines**								
A1	Pyrazine	290-37-9	1211	1214	6.48±1.20	6.26±0.10	6.87±0.29	8.62±0.24	MS/RI
A2	2-Methylpyrazine	109-08-0	1268	1270	60.00±2.26	43.72±1.17	55.55±4.02	57.85±2.73	MS/RI
A3	2,5-Dimethylpyrazine	123-32-0	1325	1326	1106.16±4.72	834.62±23.12	1107.94± 42.94	1425.37±21.40	MS/RI/O/S
A4	2,6-Dimethylpyrazine	108-50-9	1332	1334	68.20±1.90	59.65±3.04	61.98±5.39	98.82±2.32	MS/RI/O
A5	2,3-Dimethylpyrazine	5910-89-4	1350	1346	76.74±0.97	87.03±3.42	85.95±3.19	113.48±0.66	MS/RI/O/S
A6	2-Methyl-5-ethylpyrazine	13360-64-0	1372	1376	ND	143.90±43.86	138.70±44.94	13.14±0.50	MS/RI/O
A7	2-Methyl-6-ethylpyrazine	13925-03-6	1388	1390	ND	ND	ND	7.10±0.35	MS/RI
A8	2,3,5-Trimethylpyrazine	14667-55-1	1408	1408	1236.30±3.18	1291.51±8.70	1275.14± 37.96	1416.78±25.19	MS/RI/O/S
A9	3-Ethyl-2,5-dimethylpyrazine	13360-65-1	1445	1447	58.21±0.50	45.87±1.33	64.29±4.80	79.00±3.65	MS/RI/O/S
A10	5-Ethyl-2,3-dimethylpyrazine	15707-34-3	1463	1460	30.18±0.66	38.14±2.28	36.65±2.95	55.98±2.66	MS/RI
A11	2,3,5,6-Tetramethylpyrazine	1124-11-4	1477	1478	1203.61± 27.40	1708.16± 106.25	1500.72± 53.53	1908.61±67.39	MS/RI/O/S
A12	2-Ethyl-3,5,6-trimethylpyrazine	17398-16-2	1519	1491	87.79±2.26	145.20±11.15	131.96±16.61	168.89±9.76	MS/RI
A13	2-Isobutyl-3,5,6-trimethylpyrazine	46187-37-5	1595	1580	2.50±0.13	ND	ND	ND	MS/RI
B	**Alcohols**								
B1	Methanethiol	74-93-1	-	675	2.50±0.05	2.80±0.47	3.27±0.40	3.33±0.16	MS/RI
B2	Glycidol	556-52-5	1285	1286	67.78±13.66	56.21±1.48	70.09±3.37	52.72±13.64	MS/RI
B3	2-Methyl-1-propanol	78-83-1	1089	1113	ND	ND	ND	0.52±0.05	MS/RI
B4	3-Methylbut-3-en-1-ol	763-32-6	1245	1246	ND	ND	2.16±0.02	4.09±0.25	MS/RI
B5	1-Pentanol	71-41-0	1245	1241	4.52±0.13	3.76±0.37	4.38±0.17	ND	MS/RI
B6	2-Heptanol	543-49-7	1315	1316	ND	2.81±0.24	ND	ND	MS/RI
B7	1-Hexanol	111-27-3	1348	1348	169.51±2.56	159.01±11.09	172.45±0.66	1425.37±21.40	MS/RI
B8	(*Z*)-Hex-3-en-1-ol	928-96-1	1380	1377	ND	7.49±0.38	8.38±0.06	4.87±0.07	MS/RI

No.	Aroma Compounds	CAS	RI[a]	RI[b]	Compound Content (μg /kg)				Identification method[c]
					BJ3-2	BJ3-2Δ*ilvA*	BJ3-2Δ*serA*	BJ3-2Δ*serA*Δ*ilvA*	
B9	(*E*)-Hex-3-en-1-ol	544-12-7	1380	1379	9.01±0.24	ND	ND	ND	MS/RI
B10	3-Octanol	589-98-0	1388	1385	12.48±0.54	10.75±0.46	12.12±0.37	9.43±0.45	MS/RI
B11	(*E*)-Hex-2-en-1-ol	928-95-0	1400	1400	3.71±0.09	3.27±0.13	3.73±0.09	1.95±0.21	MS/RI
B12	1-Octen-3-ol	3391-86-4	1439	1438	246.01±8.79	234.51±12.55	252.83±5.66	202.68±6.48	MS/RI/O/S
B13	2,6-Dimethylheptan-4-ol	108-82-7	1469	1509	110.78±2.95	100.78±8.73	111.92±5.49	105.07±2.57	MS/RI
B14	(2R,3R)-butane-2,3-diol	24347-58-8	1537	1544	1067.78±72.34	1007.39±87.16	875.73±103.18	544.27±34.56	MS/RI
B15	1-Octanol	111-87-5	1554	1556	ND	ND	ND	1.61±0.27	MS/RI
B16	6-Undecanol	23708-56-7	1648	1640	19.72±0.32	20.06±1.99	22.58±2.46	13.30±0.93	MS/RI
B17	3-Furanmethanol	4412-91-3	1653	1679	37.60±2.13	37.75±3.45	46.44±1.40	42.11±1.91	MS/RI/O
B18	3-Methylsulfanylpropan-1-ol	505-10-2	1714	1715	ND	1.94±0.42	ND	ND	MS/RI
B19	Benzyl alcohol	100-51-6	1874	1872	10.74±0.71	9.61±1.21	12.41±1.42	12.35±1.30	MS/RI
B20	Phenylethyl Alcohol	60-12-8	1913	1912	16.39±1.72	16.38±4.25	20.94±3.02	16.90±1.95	MS/RI
C	**Ketones**								
C1	Acetone	67-64-1	809	834	130.41±3.06	125.82±13.07	132.54±2.70	80.24±1.14	MS/RI/O/S
C2	2-Butanone	78-93-3	887	905	107.30±2.06	102.34±9.66	102.39±0.97	72.41±2.78	MS/RI
C3	3-Methylbutan-2-one	563-80-4	917	929	2.80±0.26	2.90±0.53	2.41±0.07	11.25±2.12	MS/RI
C4	2,3-Butanedione	431-03-8	960	963	1146.78±27.69	1349.14±29.86	1315.87±48.02	1092.93±21.19	MS/RI/O/S
C5	2,3-Pentanedione	600-14-6	1047	1050	ND	ND	6.30±0.71	4.83±0.53	MS/RI
C6	2,3-Hexanedione	3848-24-6	1121	1138	ND	ND	0.00±0.00	2.07±0.29	MS/RI
C7	2,5-dimethylhexan-3-one	1888-57-9	1142	1145	27.62±4.86	18.15±4.27	ND	ND	MS/RI
C8	2-methylheptan-3-one	13019-20-0	1147	1164	6.40±3.07	ND	ND	36.89±1.58	MS/RI
C9	2-Heptanone	110-43-0	1181	1180	10.69±1.73	13.34±2.14	11.35±0.19	6.19±1.92	MS/RI
C10	6-Methylheptan-2-one	928-68-7	1235	1237	ND	1.11±0.13	ND	ND	MS/RI
C11	3-Methylacetoin	115-22-0	1240	1243	4.36±0.38	5.21±0.84	ND	ND	MS/RI
C12	3-Octanone	106-68-3	1255	1254	19.54±4.66	16.44±3.65	20.09±1.68	18.86±2.14	MS/RI

No.	Aroma Compounds	CAS	RI[a]	RI[b]	Compound Content (μg /kg)				Identification method[c]
					BJ3-2	BJ3-2Δ*ilvA*	BJ3-2Δ*serA*	BJ3-2Δ*serA*Δ*ilvA*	
C13	Acetoin	513-86-0	1285	1286	1029.42±56.82	1356.25±47.91	1207.23±17.81	800.73±3.66	MS/RI
C14	1-Octen-3-one	4312-99-6	1301	1308	6.73±0.35	6.00±1.25	4.83±0.22	3.98±0.11	MS/RI
C15	2,3-Octanedione	585-25-1	1319	1325	9.90±0.29	9.75±0.15	11.29±0.77	9.83±0.69	MS/RI
C16	2-Hydroxy-3-pentanone	5704-20-1	1358	1361	23.43±0.55	7.53±1.29	22.68±0.68	5.85±0.15	MS/RI
C17	Acetoin acetate	4906-24-5	1375	1378	153.62±1.58	172.77±17.11	178.59±6.85	98.55±0.85	MS/RI
C18	Cyclotene	80-71-7	1830	1830	3.12±0.29	2.73±0.33	3.59±0.32	ND	MS/RI
D	**Acids**								MS/RI
D1	Acetic acid	64-19-7	1434	1452	319.62±47.70	265.01±144.78	246.29±68.37	356.43±90.76	MS/RI
D2	2-Methylpropanoic acid	79-31-2	1562	1571	173.49±7.10	250.44±9.14	214.60±20.38	334.44±5.90	MS/RI
D3	2-Methylbutanoic acid	116-53-0	1661	1682	571.99±46.18	527.32±16.88	599.32±38.31	735.05±10.98	MS/RI/O/S
D4	4-Methylvaleric acid	646-07-1	1811	1800	ND	ND	ND	23.36±1.23	MS/RI
D5	Malic acid	6915-15-7	-	-	161.09±25.64	64.54±4.16	86.44±1.21	181.88±29.60	MS/O
E	**Aldehydes**								MS/RI
E1	Propanal	123-38-6	779	783	1.06±0.14	0.96±0.02	0.95±0.05	1.92±0.22	MS/RI
E2	2-Propenal	107-02-8	834	828	1.04±0.08	1.07±0.13	1.28±0.06	ND	MS/RI
E3	2-Methylpropanal	78-84-2	861	855	ND	0.33±0.02	ND	ND	MS/RI
E4	2-Methylbutanal	96-17-3	901	903	12.73±0.33	9.41±1.29	13.44±0.94	10.70±0.63	MS/RI
E5	3-Methylbutanal	590-86-3	904	900	24.69±1.21	17.98±2.39	24.60±1.56	21.61±0.48	MS/RI
E6	2-Butenal	4170-30-3	1034	1038	7.80±0.42	10.27±0.40	9.52±0.32	4.69±0.42	MS/RI
E7	Hexanal	66-25-1	1075	1078	9.91±4.95	9.74±4.10	10.73±2.20	4.13±1.48	MS/RI
E8	(*E*)-2-Methylbut-2-enal	1115-11-3	1091	1093	22.13±6.55	22.88±4.19	17.91±3.43	10.61±5.00	MS/RI
E9	2-Ethyl-2-hexenal	645-62-5	1336	1330	1.76±0.75	ND	8.92±6.67	ND	MS/RI
E10	Nonanal	124-19-6	1392	1390	ND	2.08±0.60	ND	4.87±2.02	MS/RI
E11	3-Furaldehyde	498-60-2	1457	1455	8.42±0.25	7.53±0.70	9.20±0.19	5.86±0.52	MS/RI
E12	Benzaldehyde	100-52-7	1524	1529	210.21±4.03	162.21±4.38	240.49±8.72	215.67±9.19	MS/RI

No.	Aroma Compounds	CAS	RI[a]	RI[b]	Compound Content (μg /kg)				Identification method[c]
					BJ3-2	BJ3-2Δ*ilvA*	BJ3-2Δ*serA*	BJ3-2Δ*serA*Δ*ilvA*	
E13	2-Phenylcrotonaldehyde	4411-89-6	1936	1922	1.93±0.11	1.79±0.06	2.62±0.23	2.63±0.25	MS/RI
E14	1H-Pyrrole-2-carbaldehyde	1003-29-8	2023	2028	3.62±0.37	3.09±0.54	4.05±0.24	4.86±0.49	MS/RI
F	**phenols**								MS/RI
F1	2-Methoxy-5-methylphenol	1195-09-1	1747	1789	3.80±0.27	8.34±1.86	7.65±1.40	ND	MS/RI
F2	Guaiacol	32994	1857	1859	69.51±1.20	84.10±5.56	96.15±9.46	117.88±8.70	MS/RI/O/S
F3	Phenol	108-95-2	1996	2000	73.65±2.50	56.08±5.97	82.77±6.11	84.20±6.37	MS/RI
G	**Esters**								
G1	Methyl ethanoate	79-20-9	820	839	13.93±0.25	16.42±0.93	17.30±3.47	9.47±1.04	MS/RI
G2	Ethyl Acetate	141-78-6	872	889	0.85±0.08	0.89±0.07	0.89±0.06	ND	MS/RI
G3	Methyl thiolacetate	1534-08-3	1040	1041	ND	3.13±1.67	ND	ND	MS/RI
G4	Hexyl acetate	142-92-7	1272	1275	ND	3.57±0.37	5.24±1.07	2.25±0.57	MS/RI
G5	Methylbenzoate	93-58-3	1626	1631	ND	ND	ND	1.03±0.10	MS/RI
G6	4-Hexanolide	695-06-7	1705	1696	6.34±0.12	5.37±0.74	7.34±0.59	ND	MS/RI
G7	(S)-Ethyl 2-hydroxy-2-phenylacetate	13704-09-1	-	-	ND	ND	2.44±0.63	ND	MS
H	**Furan**								MS/RI
H1	Furan	110-00-9	786	797	ND	ND	0.29±0.01	ND	MS/RI
H2	3-Methylfuran	930-27-8	856	851	0.63±0.04	0.64±0.09	0.71±0.03	0.32±0.11	MS/RI
H3	2-Ethylfuran	3208-16-0	938	949	17.13±2.10	17.11±3.30	15.24±2.67	4.06±0.95	MS/RI
H4	2-Ethyl-5-methylfuran	1703-52-2	1023	1018	0.71±0.09	0.65±0.38	0.66±0.08	ND	MS/RI
H5	2-n-Butyl furan	4466-24-4	1133	1123	ND	ND	1.04±0.17	ND	MS/RI
H6	2-Pentylfuran	3777-69-3	1227	1228	13.34±11.59	25.70±8.88	13.93±0.85	15.48±2.64	MS/RI
I	**Others**								
I1	1,4-Pentadiene	591-93-5	-	-	ND	ND	ND	0.19±0.07	MS
I2	Carbon disulfide	75-15-0	-	-	ND	ND	ND	0.91±0.20	MS/RI
I3	2,4,5-Trimethyl-1,3-dioxolane	3299-32-9	929	967	ND	0.88±0.10	ND	ND	MS/RI
I4	Acetonitrile	27522	988	1003	1.87±0.06	3.19±0.26	2.27±0.13	2.35±0.19	MS/RI
I5	Toluene	108-88-3	1033	1037	ND	3.99±0.37	ND	2.18±0.28	MS/RI

No.	Aroma Compounds	CAS	RI[a]	RI[b]	Compound Content (μg /kg)				Identification method[c]
					BJ3-2	BJ3-2Δ*ilvA*	BJ3-2Δ*serA*	BJ3-2Δ*serA*Δ*ilvA*	
I6	Dimethyl disulfide	624-92-0	1065	1085	1.79±0.17	1.49±0.17	1.63±0.33	1.73±0.20	MS/RI
I7	1,3-Dimethylbenzene	108-38-3	1135	1138	0.67±0.24	ND	ND	ND	MS/RI
I8	Pyridine	110-86-1	1191	1191	ND	ND	2.62±0.14	1.87±0.56	MS/RI
I9	2,4,5-trimethyloxazole	20662-84-4	1198	1200	54.35±8.53	131.74±11.65	81.32±2.53	56.49±1.79	MS/RI/O/S
I10	Pyrazinamide	98-96-4	1711	1740	9.20±0.51	5.32±0.58	8.79±0.73	6.69±0.50	MS/RI
I11	1-(1H-Pyrrol-2-yl)-ethanone	1072-83-9	1971	1971	1.69±0.17	1.38±0.29	1.89±0.16	2.68±0.34	MS/RI
I12	Unknown1	Unknown1	-	-	ND	1.68±0.00	ND	ND	MS
I13	unknown2	unknown2	-	-	140.42±19.98	64.67±31.18	103.88±11.41	99.60±18.80	MS

a: Retention indices were obtained from the flavornet database (https://webbook.nist.gov/chemistry/).

b: Retention index calculated of compounds on DB-WAX Column (60 m x 0.25 mm x 0.25 μm) with a homologous series of n-alkanes (C7-C30).

c: Identification methods: MS, RI, O and S represent being identifying by NIST 20 mass spectral database, linear retention indices reported in the species database (http://webbook.nist.gov/chemistry/name-ser.html) and literature, olfactometry, and standard chemical, respectively.

ND: Not detected

Table S5. rOAVs of the odor-active substances (rOAV>1) in four samples

No.[a]	Compounds	Threshold[b] (μg /kg)	rOAV			
			BJ3-2	BJ3-2Δ*ilvA*	BJ3-2Δ*serA*	BJ3-2Δ*serA*Δ*ilvA*
E4	2-Methylbutanal	1	12.73	9.41	13.44	10.70
H3	2-Ethylfuran	2.3	7.45	7.44	6.62	1.76
C4	2,3-Butanedione	3	382.26	449.71	438.62	364.31
I6	Dimethyl disulfide	0.06	29.79	24.90	27.16	28.77
E7	Hexanal	10	0.99	0.97	1.07	0.41
I9	2,4,5-trimethyl-1,3-oxazole	5	10.87	26.35	16.26	11.30
H6	2-Pentylfuran	4.8	2.78	5.35	2.90	3.23
C13	Acetoin	259	3.97	5.24	4.66	3.09
A3	2,5-Dimethylpyrazine	20	55.31	41.73	55.40	71.27
B7	1-Hexanol	700	0.24	0.23	0.25	2.04
A5	2,3-Dimethylpyrazine	100	0.77	0.87	0.86	1.13
E10	Nonanal	3.5	0.00	0.59	0.00	1.39
A8	2,3,5-Trimethylpyrazine	10	123.63	129.15	127.51	141.68
B12	1-Octen-3-ol	7	35.14	33.50	36.12	28.95
A9	3-Ethyl-2,5-dimethylpyrazine	25	2.33	1.83	2.57	3.16
A11	2,3,5,6-Tetramethylpyrazine	200	6.02	8.54	7.50	9.54
F2	Guaiacol	0.17	408.89	494.69	565.58	693.43
D5	Malic acid	64	2.52	1.01	1.35	2.84
D3	2-Methylbutanoic acid	520	1.10	1.01	1.15	1.41

a: The numbers assigned to the compounds are in line with those specified in TableS1.

b: Odor thresholds were referenced from the book (Odor thresholds compilations of odor threshold values in air, water and other media) and a literature.

Table S6. Odor intensity of the odorants in four samples identified using GC-O

No.[a]	Compounds	GC-O			
		BJ3-2	BJ3-2Δ*ilvA*	BJ3-2Δ*serA*	BJ3-2Δ*serA*Δ*ilvA*
D3	2-Methylbutanoic acid	3	3	3	4
A11	2,3,5,6-Tetramethylpyrazine	2	2.5	2.3	3
D5	Malic acid	3	2	2	3
A8	2,3,5-Trimethylpyrazine	3	2	3	4
A3	2,5-Dimethylpyrazine	3.1	2	3	4
A9	3-Ethyl-2,5-dimethylpyrazine	2	2	2	3
F2	Guaiacol	2.3	2	3	4
C4	2,3-Butanedione	2	3	3	2
A10	5-Ethyl-2,3-dimethylpyrazine	2	2	2	2.8
A5	2,3-Dimethylpyrazine	2	2	2	3
C13	Acetoin	2	3	3	2
D2	2-Methylpropanoic acid	2	2	2	3
B12	1-Octen-3-ol	2	2	2	2
B17	3-Furanmethanol	2	2	2	2
A4	2,6-Dimethylpyrazine	1	1	1	2
I9	2,4,5-trimethyl-1,3-oxazole	1	3	1	1
A6	2-Methyl-5-ethylpyrazine	1	1	1	1

a: The numbers assigned to the compounds are in line with those specified in TableS1.
