# Supplementary material for: Characterization of Key Aroma Compounds of Soy Sauce-like Aroma Produced in Ferment of Soybeans by Bacillus subtilis BJ3-2
Source: Foods. 2024 Aug 28;13(17):2731. doi: 10.3390/foods13172731 (PMC11395551; doi:10.3390/foods13172731)
Supplement: Supplementary file 1 [file foods-13-02731-s001.zip › Supplementary file S2.pdf]

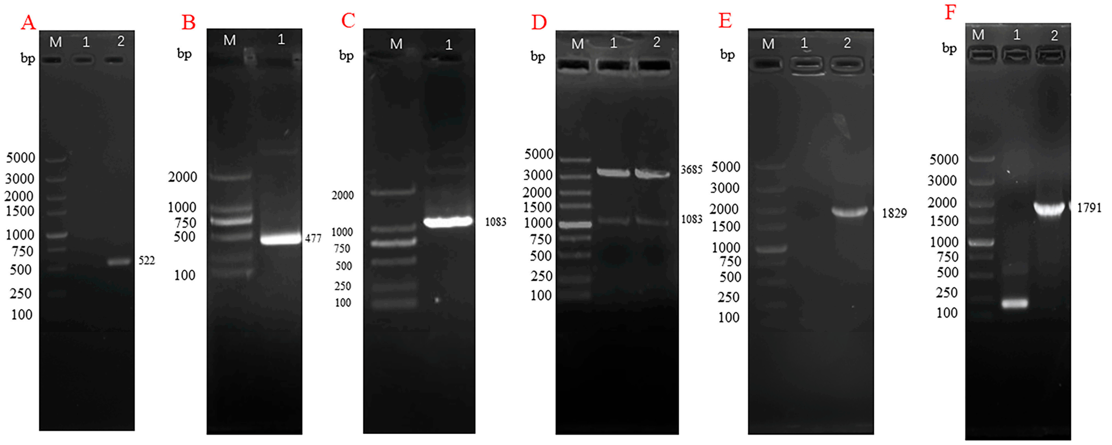

Figure S1. ***ilvA*** **homologous recombination knockout vector construction and transformation of the vector. A** Amplification of HLarm (M: DL5000marker, line 1: blank, line 2: HLarm). **B** Amplification of HRarm (M: DL5000marker, line 1: blank, line 2: HRarm). **C** Amplification of *cat* (M: DL2000marker, line 1: *cm*). **D** Double digestion of the knockout vector by *Kpn* I and *sal* I (M: DL5000marker, line 1-2: double digestion). **E** Double-exchange PCR validation of *ilvA* DEDP F and cm R (M: DL5000marker, line 1: BJ3-2, line 2: transformants). **F** Double-exchange PCR validation of *ilvA cm* F and DEDP R (M: DL5000marker, line 1: BJ3-2, line 2: transformants).

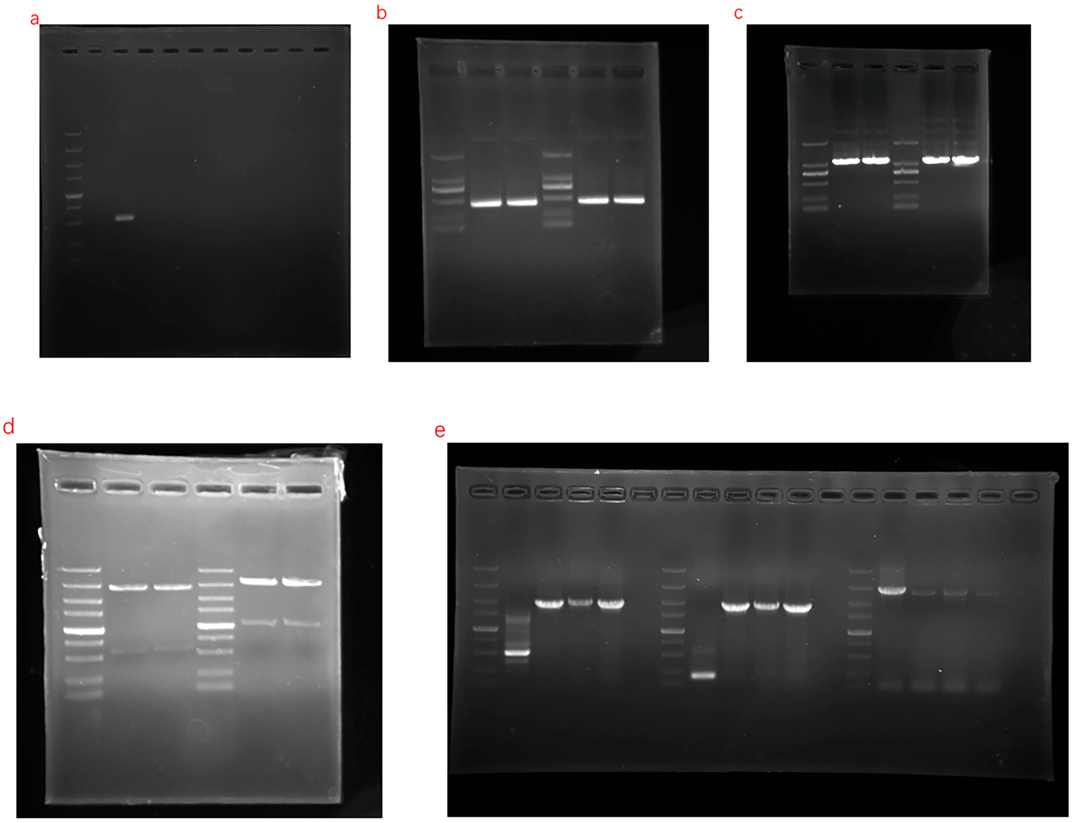

a is the original diagram of Figure S1A. **b** is the original diagram of Figure S1B. **c** is the original diagram of Figure S1C. **d** is the original diagram of Figure S1D. **e** is the original diagrams of Figure S1E and F.

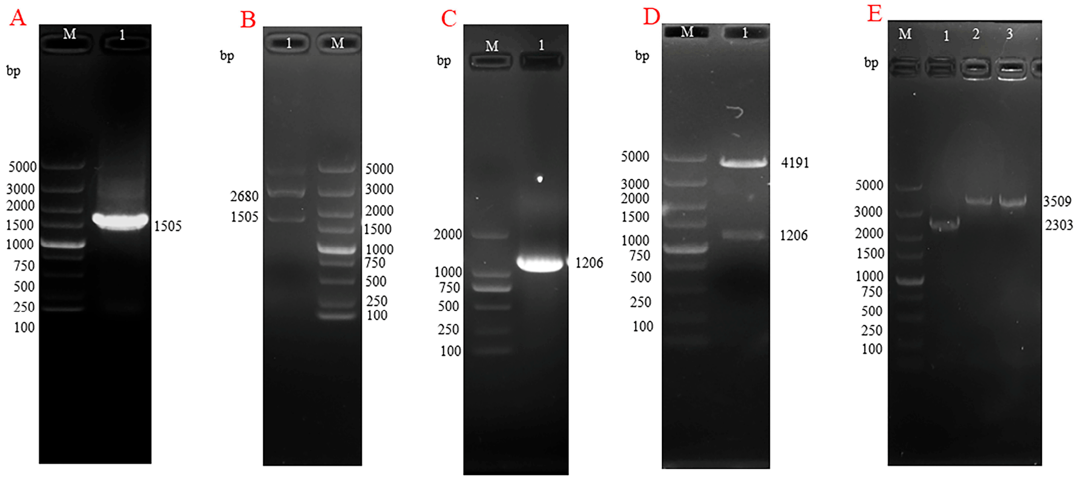

Figure S2. ***serA*** **homologous recombination knockout vector construction and transformation of the vector. A** Amplification of *serA* (M: DL5000marker, line 1: *serA*). **B** Double digestion of the knockout vector by *Sal I* and *Sph I* (M: DL5000marker, line 1: double digestion). **C** Amplification of *erm* (M: DL2000marker, line 1: *erm*). **D** Digestion of the knockout vector by *Pst* I (M: DL5000marker, line 1: digestion). **E** Double-exchange PCR validation of *serA* DEDP F and DEDP R (M: DL5000marker, line 1: BJ3-2, line 2 : transformants).

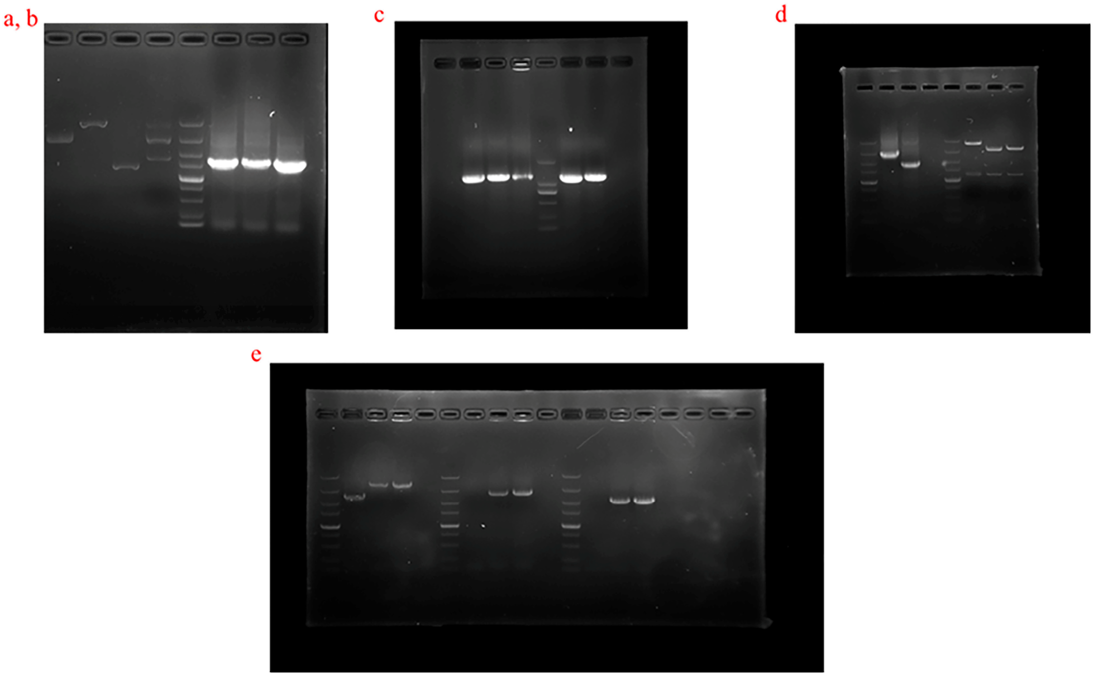

a,b is the original diagram of Figure S1A and B. **c** is the original diagram of Figure S1C. **d** is the original diagram of Figure S1D. **e** is the original diagram of Figure S1E.

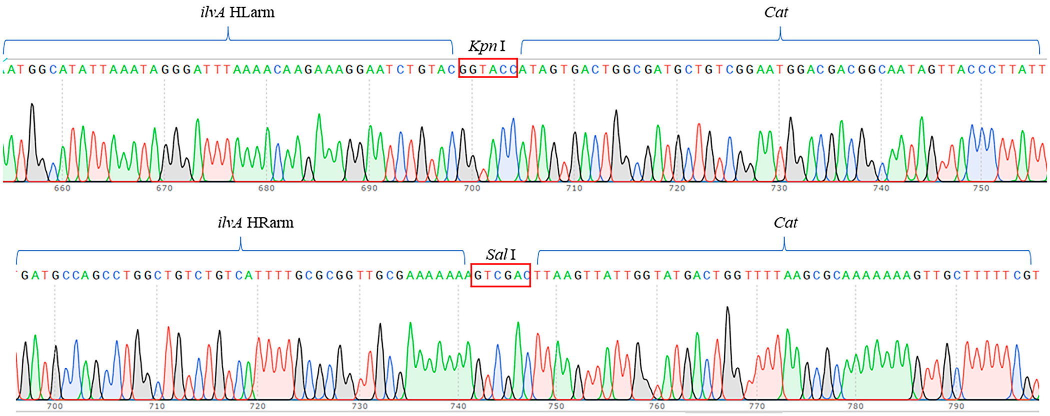

Figure S3. Sequencing of BJ3-2Δ*ilvA*.

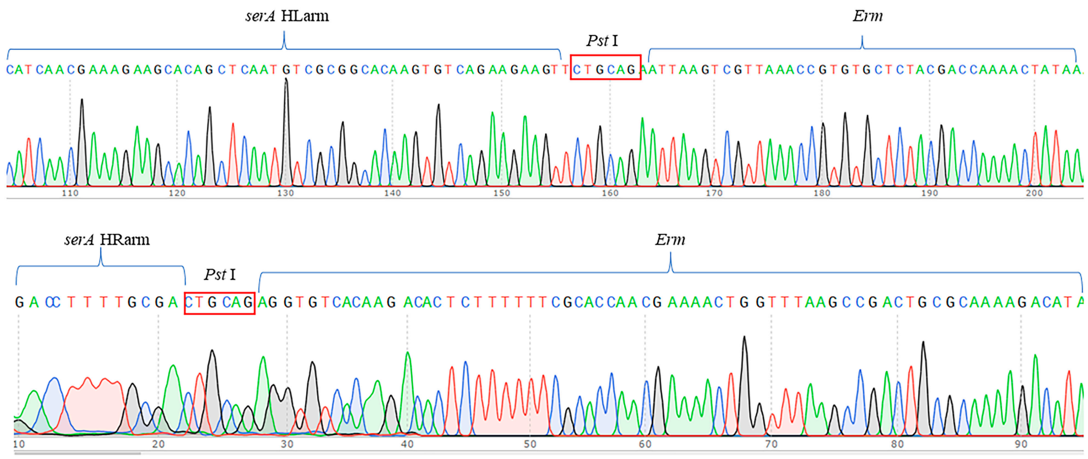

Figure S4. Sequencing of BJ3-2Δ*serA*.

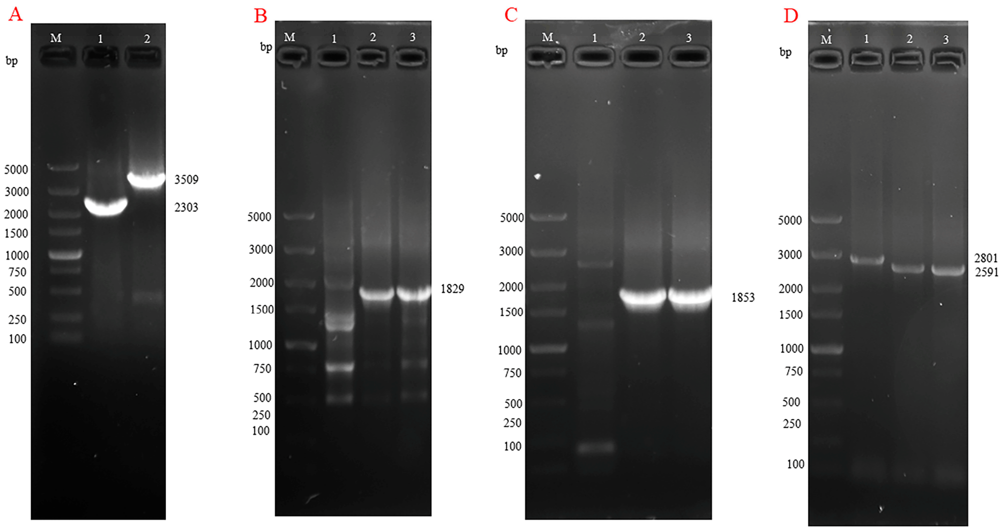

Figure S5. PCR validation of BJ3-2Δ*serA*Δ*ilvA*. A Double-exchange PCR validation by *serA* DEDP2 F and *serA* DEDP2 R (M: DL5000 Marker, lane 1: BJ3-2, lane 2: transformant). **B** Double-exchange PCR validation of *ilvA* DEDP F and *cat* R (M: DL5000 Marker, lane 1: BJ3-2Δ*serA*, lane 2-3: transformants). **C** d Double-exchange PCR validation of *cat* F and *ilvA* DEDP R (M: DL5000 Marker, lane 1: BJ3-2Δ*serA*, lane 2-3: transformants). **D** Double-exchange PCR validation of *ilvA* DEDP F and *ilvA* DEDP R (M: DL5000 Marker, lane 1: BJ3-2Δ*serA*, lane 2-3: transformants).

[illegible]

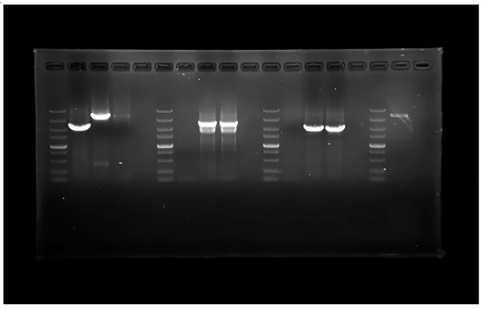

b, c, d

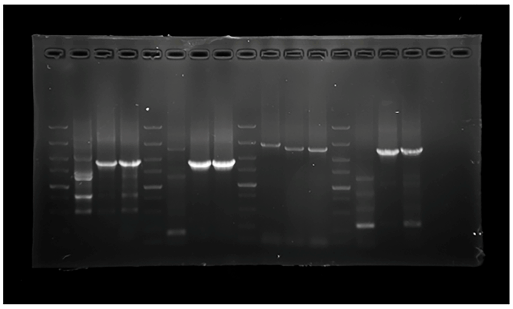

a is the original diagram of Figure S5A. **b**, **c**, **d** is the original diagram of Figure S5B, Cand D.

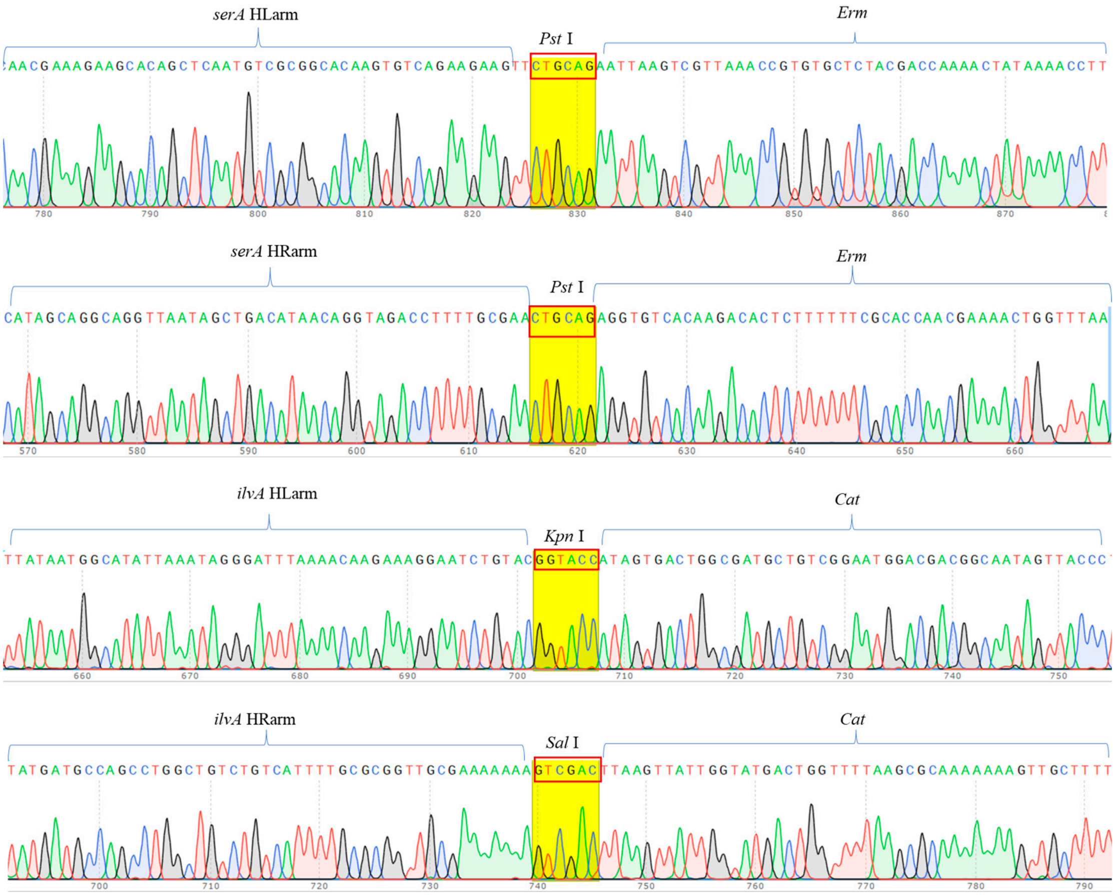

Figure S6. Sequencing of BJ3-2Δ*serA*Δ*ilvA*.
